# Systematic Dementia Screening by Multidisciplinary Team Meetings in Nursing Homes for Reducing Emergency Department Transfers: The IDEM Cluster Randomized Clinical Trial

**DOI:** 10.1001/jamanetworkopen.2020.0049

**Published:** 2020-02-26

**Authors:** Yves Rolland, Neda Tavassoli, Philipe de Souto Barreto, Amélie Perrin, Clarisse Laffon de Mazières, Thomas Rapp, Sophie Hermabessière, Elodie Tournay, Bruno Vellas, Sandrine Andrieu

**Affiliations:** 1 Gérontopôle de Toulouse, Département de Médecine Interne et Gérontologie Clinique, Centre Hospitalo-Universitaire de Toulouse, Toulouse, France; 2 Équipe Régionale Vieillissement et Prévention de la Dépendance (ERVPD), Gérontopôle de Toulouse, Centre Hospitalo-Universitaire de Toulouse, Toulouse, France; 3 UMR 1027, INSERM–Université de Toulouse III, Toulouse, France; 4 LIRAES (EA 4470) & Chaire AGEINOMIX, Université Paris Descartes Sorbonne Paris Cité, Paris, France; 5 Unité de Soutien Méthodologique à la Recherche, Centre Hospitalier Universitaire de Toulouse, Toulouse, France; 6 Service d'Epidémiologie, Unité de Soutien Méthodologique à la Recherche, Centre Hospitalier Universitaire de Toulouse, Toulouse, France

## Abstract

**Importance:**

Dementia is often underdiagnosed in nursing homes (NHs). This potentially results in inappropriate care, and high rates of emergency department (ED) transfers in particular.

**Objective:**

To assess whether systematic dementia screening of NH residents combined with multidisciplinary team meetings resulted in a lower rate of ED transfer at 12 months compared with usual care.

**Design, Setting, and Participants:**

Multicenter, cluster randomized trial with NHs as the unit of randomization. The IDEM (Impact of Systematic Tracking of Dementia Cases on the Rate of Hospitalization in Emergency Care Units) trial took place at 64 public and private NHs in France. Recruitment started on May 1, 2010, and was completed on March 31, 2012. Residents who were aged 60 years or older, had no diagnosed or documented dementia, were not bedridden, had lived in the NH for at least 1 month at inclusion, and had a life expectancy greater than 12 months were included. The residents were followed up for 18 months. The main study analyses were completed on October 14, 2016.

**Intervention:**

Two parallel groups were compared: an intervention group consisting of NHs that set up 2 multidisciplinary team meetings to identify residents with dementia and to discuss an appropriate care plan, and a control group consisting of NHs that continued their usual practice. During the inclusion period of 23 months, all residents of participating NHs who met eligibility criteria were included in the study.

**Main Outcomes and Measures:**

The primary end point (ED transfer) was analyzed at 12 months, but the residents included were followed up for 18 months.

**Results:**

A total of 64 NHs participated in the study and enrolled 1428 residents (mean [SD] age, 84.7 [8.1] years; 1019 [71.3%] female): 599 in the intervention group (32 NHs) and 829 in the control group (32 NHs). The final study visit was completed by 1042 residents (73.0%). The main reason for early discontinuation was death (318 residents [22.7%]). The intervention did not reduce the risk of ED transfers during the 12-month follow-up: the proportion of residents transferred at least once to an ED during the 12-month follow-up was 16.2% in the intervention group vs 12.8% in the control group (odds ratio, 1.32; 95% CI, 0.83-2.09; *P* = .24).

**Conclusions and Relevance:**

This study failed to demonstrate that systematic screening for dementia in NHs resulted in fewer ED transfers. The findings do not support implementation of multidisciplinary team meetings for systematic dementia screening of all NH residents, beyond the national recommendations for dementia diagnosis, to reduce ED transfers.

**Trial Registration:**

ClinicalTrials.gov Identifier: NCT01569997

## Key Points

**Question:**

Does systematic dementia screening of nursing home residents combined with multidisciplinary team meetings result in a lower rate of emergency department transfer compared with usual care?

**Findings:**

In this cluster randomized clinical trial that included 1428 residents in 64 nursing homes, 16.2% of residents in the intervention group were transferred to emergency departments vs 12.8% in the control group during the 12-month follow-up, a nonsignificant difference.

**Meaning:**

This study does not suggest that systematic screening for dementia in all nursing home residents reduces emergency department transfers.

## Teaser

This cluster randomized trial evaluates the effect of dementia screening with multidisciplinary team meetings on the rate of transfer to emergency departments for nursing home residents in France.

## Introduction

In populations with multiple underlying morbidities, such as nursing home (NH) residents, dementia is highly prevalent and frequently associated with potentially avoidable adverse events, including falls, weight loss, delirium, side effects of polymedication, and behavioral disturbances. Although systematic screening for dementia in NH residents^1^ is recommended by expert groups and by guidelines, underrecognition of the disease has been repeatedly reported in Europe and in the United States^2^ and has been identified as an indicator of poor-quality care.^3^ Underdiagnosis of dementia has resulted in inappropriate health care^4,5,6,7^ and, in particular, a high hospitalization rate, notably emergency department (ED) admissions. However, to our knowledge, the hypothesis that dementia screening in NH residents results in a lower ED transfer rate has never been demonstrated.

The aim of this cluster randomized clinical trial was to assess whether systematic dementia screening in NH residents, combined with multidisciplinary team meetings (MDTMs), resulted in a lower ED transfer rate compared with usual care.

## Methods

The IDEM (Impact of Systematic Tracking of Dementia Cases on the Rate of Hospitalization in Emergency Care Units) study was a multicenter study cluster randomized by NH (1:1) that compared 2 groups: an intervention group consisting of NHs that set up MDTMs to identify residents with dementia and to propose an appropriate care plan, and a control group of NHs that continued their usual practice. The cluster randomized design was chosen for this study because it was difficult to include residents in both intervention and control groups in the same NH without the risk of contamination between the 2 groups. Recruitment started on May 1, 2010, and was completed on March 31, 2012. The residents were followed up for 18 months. The main study analyses were completed on October 14, 2016. The study protocol (available in Supplement 1) was approved by the French Ethics Committee for the Protection of Persons and the competent authority located in Toulouse. Oral informed consent for study participation was obtained from all residents or their representatives by the NH coordinating physicians (more details about the role of the coordinating physician appear in eAppendix 1 in Supplement 2). Participants’ written informed consent was not required by French law at the time of the study. This study followed the Consolidated Standards of Reporting Trials (CONSORT) reporting guideline.

### Cluster Randomization

The unit of randomization was the NH. Before starting resident recruitment, cluster randomization with a 1:1 ratio was performed using STATA software version 9 (StataCorp LP) to allocate NHs to the intervention or the control group (stratification criteria appear in eAppendix 2 in Supplement 2).

### Inclusion and Exclusion Criteria

Nursing homes located in various regions of France participated in the study on a voluntary basis. There were no exclusion criteria for nursing home participation. When the project was set up, the coordinating physician of each NH designated a memory clinic in the same area where the MDTMs could be held with the NH staff.

During the inclusion period, all the residents of participating NHs who met the study criteria were included in the study: residents aged 60 years or older, without diagnosed or documented dementia, not bedridden (Groupe Iso-Ressources [GIR] score >1; GIR is the French level-of-dependence score from 1 to 6, where 1 indicates completely dependent or bedridden and 6 indicates completely independent),^8^ living in the NH for at least 1 month at inclusion, with a life expectancy of more than 1 year, and without any disease likely to jeopardize his or her participation in the study.

### Inclusion and Visits in NHs

After a 3-month preselection period, each coordinating physician participated in an inclusion visit with all the NH’s eligible residents over a period of 2 months. Sociodemographic and medical data were collected by the coordinating physician in both groups during the inclusion visit. The residents in the intervention group also underwent a comprehensive geriatric assessment. The residents included were followed up for 18 months. At the end of follow-up, all residents in both groups underwent a final visit in the NH with the coordinating physician including a simplified comprehensive geriatric assessment (the tests performed at the inclusion and final visits are described in eAppendix 4 in Supplement 2).

### Intervention: MDTMs

In the intervention group, 2 MDTMs were organized for each NH with its associated memory clinic where the records of all participating residents were analyzed. The first meeting took place in the first month after the 2-month period of recruitment and the second meeting before the 12th month of the resident’s follow-up. The case of each resident was thus discussed twice at an interval of approximately 1 year. The details of the MDTM organization and subjective qualitative assessment of the meetings have been reported elsewhere^9^ and appear in eAppendix 5 in Supplement 2.

### Outcomes

The primary efficacy outcome as prespecified in the protocol was ED transfer within the first 12 months of follow-up. The data for the primary outcome were collected monthly by the NH coordinating physicians and were entered in the IDEM electronic case report form. Transfers to the ED during the 18-month follow-up were analyzed as a secondary outcome. Other secondary outcomes were the proportion of residents with at least 1 hospital admission judged by experts as inappropriate at 18-month follow-up (these data were obtained in a subset of the total population for whom a hospitalization report was available) and the incidence rate of ED transfers during the 12 months and 18 months of follow-up for 100 person-years. To assess the impact of systematic dementia screening on the appropriateness of hospitalizations, hospitalizations were classified as appropriate or inappropriate using a standardized procedure (eAppendix 6 in Supplement 2).

### Changes to the Protocol

Owing to difficulties in including a sufficient number of participants in the study, the protocol was amended as follows: (1) the intensity of intervention on the primary outcome was increased by addition of a second MDTM and (2) the duration of residents’ follow-up was extended from 12 months to 18 months to better clarify the effect of the second MDTM on ED transfers after 18 months of follow-up as a secondary outcome. The amendments were validated by the French Ethics Committee for the Protection of Persons and the competent authority in Toulouse.

### Statistical Analysis

The number of participants needed to meet our main objective was calculated by hypothesizing a bilateral test with an α risk of 5% and a β risk of 20% (80% power). Based on previous data,^10^ we estimated the incidence of ED admissions at 24% at 12 months in the control group. To detect a 30% reduction in ED transfer rate in the intervention group, with a 2-tailed test and an α risk of 5% and 80% power, 1000 participants were required in each group, taking into account an intracluster correlation coefficient of 0.02, a 20% attrition rate, and an inflation factor related to contamination between groups of 5% to 10% during a 12-month trial. By estimating a mean number of 30 inclusions per NH, 35 institutions per group were needed.

The modified intention-to-treat population, defined as all residents included who were followed up for at least 30 days, was used as the main analysis population for all efficacy end points (primary and secondary).

Descriptive statistics were presented as mean and standard deviation or absolute numbers and percentages, as appropriate. Owing to the design of the study (randomized by cluster), regression analyses at individual level that took between-cluster variation into account were performed to assess the intervention effects. For primary efficacy analysis, ED transfers during the first 12 months of follow-up were described and compared between groups using a random-effects logistic regression model, with group as the fixed effect and NH as the random effect. The intervention effect vs control was estimated by the odds ratio and its 95% confidence interval. For participants who did not complete the 12-month follow-up, the primary end point was considered as negative if no ED transfer was reported before study termination.

For the secondary outcomes, random effects logistic regression models were used to test the intervention effect on proportions. The number of times that residents were hospitalized during follow-up was modeled by random-effects negative binomial regression, and intervention effect was measured by rate ratio with its 95% confidence interval. All secondary efficacy analyses were performed at a significance level of 5% without adjustment for multiplicity (see per-protocol and exploratory analyses in eAppendix 7 and eAppendix 8 in Supplement 2).

All analyses were performed using SAS statistical software version 9.4 (SAS Institute Inc). Significance was set at 2-tailed *P* < .05.

## Results

### Descriptive Results

Eighty-nine NHs agreed to participate in the IDEM study. After cluster randomization, 44 NHs were randomized to the intervention group and 45 to the control group. However, 25 NHs (12 in the intervention group and 13 in the control group) withdrew from the study before the recruitment of the first resident (Figure). A final total of 64 randomized NHs participated in the study, 32 in the intervention group and 32 in the control group (Table 1). Overall, during the 23-month inclusion period, 1428 residents were enrolled in the study (mean [SD] age, 84.7 [8.1] years; 1019 [71.3%] female), 599 in the intervention group and 829 in the control group. In all, 1166 residents (81.7%) were followed for at least 12 months, and the final study visit at 18 months was completed by 1042 residents (73.0%); completion rates were similar between groups. The main reason for early discontinuation was death (318 residents [22.7%]) (Figure). The mean (SD) number of months of follow-up was 15.8 (4.6) in both groups.

**Figure.**
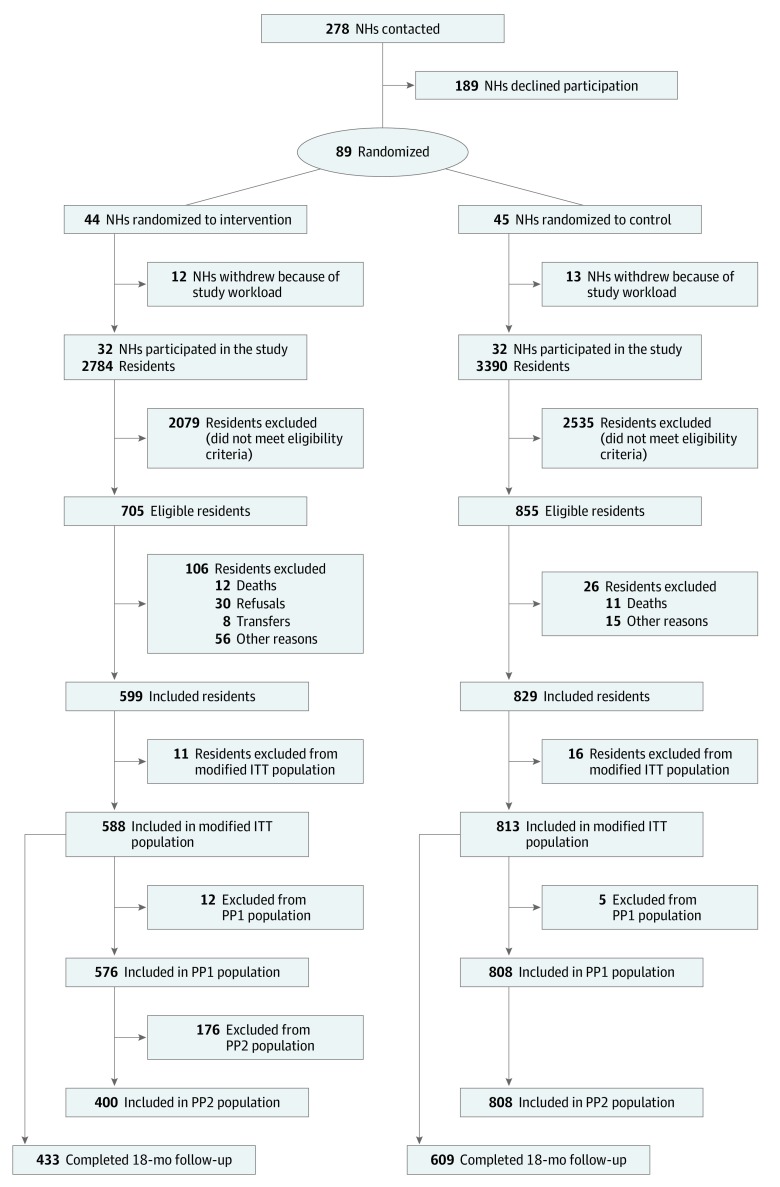
Study Flowchart Additional detail is shown in the eFigure in Supplement 2. ITT indicates intention-to-treat; NH, nursing home; and PP, per-protocol.

**Table 1.** Nursing Home Characteristics

Characteristic	Mean (SD)		
	Intervention (n = 32)	Control (n = 32)	Total (N = 64)
Stratification factors at inclusion			
Presence of specialized Alzheimer disease care unit, No. (%)	13 (40.6)	14 (43.8)	27 (42.2)
Participation of nursing home investigator in REHPA geriatric network congresses, No. (%)	29 (90.6)	25 (78.1)	54 (84.4)
GMP score	683.4 (137.44)	707.8 (96.33)	695.6 (118.37)
Description of cluster sizes			
No. of residents in the center at inclusion	81.5 (35.17)	99.7 (38.78)	90.6 (37.85)
No. of residents included by center	18.7 (8.71)	25.9 (13.56)	22.3 (11.87)
No. of residents included by center in intent-to-treat population	18.4 (8.62)	25.4 (12.99)	21.9 (11.49)
No. of residents included by center in per-protocol population 1^a^	18.0 (8.43)	25.3 (12.82)	21.6 (11.37)
No. of residents included by center in per-protocol population 2^b^	16.0 (7.33)	25.3 (12.82)	21.2 (11.64)

Abbreviations: GMP, GIR moyen pondéré (nursing home dependence score); REHPA, Recherche en Etablissement d’Hébergement pour Personnes Agées (geriatric research network in nursing homes in the Toulouse area).

^a^ Included all participants in the modified intention-to-treat population who met all eligibility criteria; the first multidisciplinary team meeting was held for participants in the intervention group.

^b^ Included all participants in the modified intention-to-treat population who met all eligibility criteria; the first and second multidisciplinary team meetings were held for participants in the intervention group.

Recruitment started on May 1, 2010, and was completed on March 31, 2012. Twenty-seven residents were excluded from the efficacy analysis: 11 in the intervention group (1.8%) and 16 in the control group (1.9%) who were followed up for less than 30 days according to the modified intention-to-treat definition. Baseline characteristics of the 1401 residents included in the modified intention-to-treat analysis (588 in the intervention group and 813 in the control group) are shown in Table 2. The results of the comprehensive geriatric assessment at inclusion and at 18-month follow-up are shown in eTable 1 in Supplement 2.

**Table 2.** Participant Characteristics at Inclusion (Modified Intention-to-Treat Population)

Characteristic	No. (%)		
	Intervention (n = 588)	Control (n = 813)	All (N = 1401)
Demographic			
Age, mean (SD), y	85.0 (7.95)	84.4 (8.19)	84.7 (8.09)
Female	422 (71.8)	582 (71.6)	1004 (71.7)
Marital status			
Married	37 (6.3)	69 (8.6)	106 (7.6)
Widowed	371 (63.2)	476 (59.3)	847 (60.9)
Single	133 (22.7)	196 (24.4)	329 (23.7)
Divorced	46 (7.8)	62 (7.7)	108 (7.8)
Education			
No education	26 (4.4)	52 (6.5)	78 (5.6)
Primary school	173 (29.5)	225 (27.9)	398 (28.6)
Primary school certificate	208 (35.4)	256 (31.8)	464 (33.3)
Elementary school or vocational diploma	105 (17.9)	158 (19.6)	263 (18.9)
A levels or higher diploma	75 (12.8)	115 (14.3)	190 (13.6)
French as native language	553 (94.0)	757 (93.1)	1310 (93.5)
Length of stay in the nursing home at inclusion, mean (SD), mo	47.7 (68.95)	56.5 (75.88)	52.8 (73.18)
Medical history			
Charlson Comorbidity Index score, mean (SD)	2.0 (1.84)	2.0 (1.99)	2.0 (1.92)
Current smoker	32 (5.4)	37 (4.6)	69 (4.9)
Alcohol consumption	188 (32.0)	227 (27.9)	415 (29.6)
Vascular risk factors	483 (82.1)	676 (83.3)	1159 (82.8)
History of psychological disorders	326 (55.5)	527 (64.9)	853 (61.0)
History of fracture	242 (41.2)	366 (45.1)	608 (43.5)
Progressive cognitive decline	123 (20.9)	167 (20.6)	290 (20.7)
Mini-Mental State Examination carried out in the past 6 mo	275 (46.9)	474 (58.4)	749 (53.6)
Mini-Mental State Examination score in the past 6 mo, mean (SD)	22.1 (5.29)	22.8 (5.22)	22.5 (5.25)
Family history of dementia	29 (4.9)	28 (3.4)	57 (4.1)
≥1 Hospital admission in past 3 mo	77 (13.1)	95 (11.7)	172 (12.3)
≥1 Emergency department admission in past 3 mo	48 (8.2)	48 (5.9)	96 (6.9)
Medication use at inclusion			
Neuroleptics	86 (14.6)	143 (17.6)	229 (16.3)
Benzodiazepines	310 (52.7)	441 (54.2)	751 (53.6)
Anxiolytics	220 (37.4)	353 (43.4)	573 (40.9)
Antidepressants	238 (40.5)	382 (47.0)	620 (44.3)
Hypnotics	198 (33.7)	286 (35.2)	484 (34.5)
Thymoregulators	30 (5.1)	62 (7.6)	92 (6.6)
Psychostimulants	238 (40.5)	384 (47.2)	622 (44.4)
Antiepileptics	84 (14.3)	125 (15.4)	209 (14.9)
Vitamin D	27 (4.6)	37 (4.6)	64 (4.6)
Vitamin K antagonists	28 (4.8)	25 (3.1)	53 (3.8)
Platelet aggregation inhibitors	214 (36.4)	293 (36.0)	507 (36.2)
Analgesics	277 (47.1)	369 (45.4)	646 (46.1)
Proton pump inhibitors	236 (40.1)	332 (40.8)	568 (40.5)
Nonmedicinal treatments			
Physiotherapy	245 (41.7)	261 (32.1)	506 (36.1)
Ergotherapy	46 (7.8)	44 (5.4)	90 (6.4)
Psychomotricity	37 (6.3)	38 (4.7)	75 (5.4)
Psychological follow-up	146 (24.8)	224 (27.6)	370 (26.4)
Dietetic follow-up	91 (15.5)	112 (13.8)	203 (14.5)
Speech therapy	10 (1.7)	5 (0.6)	15 (1.1)
Other nonmedicinal treatment	30 (5.1)	62 (7.6)	92 (6.6)

In the intervention group, the cases of 581 residents (98.8%) were presented at the first MDTM and a conclusion on dementia diagnosis was provided for 574 residents (97.6%): high probability of dementia for 129 residents (22.5%) and suspicion of dementia for 107 residents (18.6%). In 462 cases (79.5%) presented at the first MDTM, the residents’ general practitioners (GPs) were informed of the conclusion of the first MDTM, and they followed the recommendations for 61.3% of residents. The cases of 401 residents (68.2%) were also discussed in the second MDTM; the GPs of 95.9% of residents were informed of its conclusion, and for 66.6% the recommendations from the second MDTM were followed (Table 3).

**Table 3.** First and Second MDTMs in Modified Intention-to-Treat Population, Intervention Group

Characteristic	No./Total No. (%) (N = 588)^a^
First MDTM	
Residents whose case was studied during the first MDTM	581/588 (98.8)
Time spent on each case, mean (SD), min	15.8 (7.0)
No. of experts participating in the first MDTM, mean (SD)	4.2 (1.6)
Conclusion on dementia diagnosis	574/581 (98.8)
High probability of dementia	129/574 (22.5)
Absence of dementia with normal cognitive test results	207/574 (36.1)
Absence of dementia with abnormal cognitive test results	103/574 (17.9)
Suspicion of dementia	107/574 (18.6)
Lack of data or incomplete medical file	28/574 (4.9)
Symptoms present at inclusion visit	581/581 (100)
Delirium syndrome	8/581 (1.4)
Depressive syndrome	243/581 (41.8)
Malnutrition	141/581 (24.3)
High risk of fracture	272/581 (46.8)
Behavioral disturbances	153/581 (26.3)
Care plan proposed at the first MDTM	580/581 (99.8)
Proposal for further examinations	308/580 (53.1)
Laboratory tests	207/308 (67.2)
Cerebral computed tomography	101/308 (32.8)
Cerebral magnetic resonance imaging	27/308 (8.8)
Additional psychometric evaluation	113/308 (36.7)
Therapeutic proposal	470/580 (81.0)
Discussion of value of specific treatment of Alzheimer disease	64/470 (13.6)
Discontinuation of a psychotropic treatment	115/470 (24.5)
Introduction of a psychotropic treatment	8/470 (16.6)
Other changes in drug treatment	159/470 (33.8)
Proposal for nondrug treatment	271/470 (57.7)
Proposal of general preventive measures	355/470 (75.5)
Need for immediate hospitalization	29/580 (5.0)
Need for move to another nursing home	3/580 (0.5)
Resident's GP informed of MDTM conclusions	462/581 (79.5)
First MDTM recommendations followed by resident's GP	273/445 (61.3)
Second MDTM	
Residents whose case was studied during the second MDTM	401/588 (68.2)
Appearance of new developments since the first MDTM	210/401 (52.4)
Care plan proposed at the second MDTM	400/401 (99.8)
Proposal for further examinations	105/400 (26.3)
New therapeutic proposal	161/400 (40.3)
Discussion of the value of a specific treatment of Alzheimer disease	23/161 (14.3)
Discontinuation of a psychotropic treatment	64/161 (39.8)
Introduction of a psychotropic treatment	25/161 (15.5)
Other drug modifications	112/161 (69.6)
Specific recommendations to limit the need for emergency department	91/400 (22.8)
Need for regular monitoring by the memory clinic	21/91 (23.1)
Need for an outpatient specialist visit	80/91 (87.9)
Need for immediate hospitalization	6/91 (6.6)
Need for move to another nursing home	2/91 (2.2)
Resident's GP informed of second MDTM conclusions	351/366 (95.9)
Second MDTM recommendations followed by resident's GP	235/353 (66.6)
First and second MDTM recommendations followed by resident's GP	147/371 (39.6)

Abbreviations: GP, general practitioner; MDTM, multidisciplinary team meeting.

^a^ Number of participants whose data were available.

### Results of Primary Efficacy Outcome

The intervention effects on primary and secondary end point measures in the modified intention-to-treat analyses are presented in Table 4. The proportion of residents with at least 1 ED transfer in the 64 NHs during the first 12 months of follow-up showed great variation, from 0% to 58.8% among intervention NHs and from 0% to 39.3% in control NHs. The primary end point, or the proportion of residents with at least 1 ED transfer during the 12-month follow-up, was 16.2% in the intervention group and 12.8% in the control group (odds ratio, 1.32; 95% CI, 0.83-2.09; *P* = .24).

**Table 4.** Assessment of Intervention Effect on Primary and Secondary End Points in Modified Intention-to-Treat Population, Per-protocol Population 1, and Per-protocol Population 2

End Point	No./Total No. (%)		Ratio (95% CI)	*P* Value
	Intervention	Control		
Modified intention-to-treat population, No. (n = 1401)	588	813		
Primary end point: ED transfer during 12-mo follow-up^a^	95 (16.2)	104 (12.8)	1.32 (0.83-2.09)^b^	.24
Incidence rate of ED transfer during 12-mo follow-up for 100 person-years (95% CI)	20.06 (14.34-28.06)	16.27 (11.77-22.49)	1.23 (0.78-1.94)^c^	.36
ED transfer during 18-mo follow-up	118 (20.1)	145 (17.8)	1.16 (0.71 to1.91)^b^	.54
Incidence rate of ED transfer during 18-mo follow-up for 100 person-years (95% CI)	18.97 (13.55-26.55)	16.87 (12.26-23.23)	1.12 (0.71-1.77)^c^	.61
ED transfer judged inappropriate by experts during 18-mo follow-up (n = 404)	46/204 (22.5)	15/200 (7.5)	3.60 (1.90-6.84)^b^	<.001
Incidence rate of ED transfer whatever the hospital unit during 18-mo follow-up in person-years (95% CI)	73.56 (58.95-91.79)	74.43 (60.59-91.43)	0.99 (0.73-1.33)^c^	.93
Subgroup analyses: ED transfer during 12-mo follow-up in the subgroups				
NHs with Alzheimer disease unit (n = 558)	28/233 (12.0)	52/325 (16.0)	0.75 (0.41-1.41)^b^	.37
NHs without Alzheimer disease unit (n = 843)	67/355 (18.9)	52/488 (10.7)	1.88 (1.01-3.52)^b^	.04
Public NHs (n = 781)	59/402 (14.7)	55/379 (14.5)	1.00 (0.53-1.90)^b^	.99
Private NHs (n = 620)	36/186 (19.4)	49/434 (11.3)	1.85 (0.97-3.55)^b^	.06
Per-protocol population 1, No. (n = 1384)	576	808		
ED transfer during 12-mo follow-up	94 (16.3)	103 (12.7)	1.34 (0.84-2.13)^b^	.21
Incidence rate of ED transfer during 12-mo follow-up for 100 person-years (95% CI)	20.14 (14.38-28.22)	16.27 (11.76-22.52)	1.24 (0.79-1.95)^c^	.35
ED transfer during 18-mo follow-up	116 (20.1)	144 (17.8)	1.17 (0.72-1.91)^b^	.52
Incidence rate of ED transfer during 18-mo follow-up for 100 person-years (95% CI)	19.04 (13.61-26.65)	16.95 (12.32-23.32)	1.12 (0.71-1.77)^c^	.61
Per-protocol population 2, No. (n = 1208)	400	808		
ED transfer during 18-mo follow-up	71 (17.8)	144 (17.8)	0.99 (0.57-1.73)^b^	.97
Incidence rate of ED transfer during 18-mo follow-up for 100 person-years (95% CI)	14.29 (9.41-21.70)	16.65 (11.84-23.41)	0.86 (0.51-1.46)^c^	.57

Abbreviations: ED, emergency department; NH, nursing home.

^a^ The intracluster correlation coefficient indicates the similarity of measurements of participants from the same cluster with those from different clusters and was estimated at 0.1129 for the primary end point.

^b^ Odds ratio for intervention vs control.

^c^ Rate ratio for intervention vs control.

### Results of Analysis of the Secondary End Point

The secondary end point of ED transfer during the entire 18-month follow-up showed no significant differences between groups. Similar results were obtained when adjusting for confounding factors (Table 4). Regarding inappropriate hospitalizations, only hospitalizations for 404 participants for whom a report was available (204 in the intervention group and 200 in the control group) were reviewed by experts. The probability of at least 1 inappropriate hospitalization was significantly higher in the intervention group than in the control group (22.5% vs 7.5%; odds ratio, 3.60 [95% CI, 1.90-6.84]) (Table 4).

The incidence rate of ED transfer during the first 12 months of follow-up for 100 person-years (taking multiple hospitalizations into account) was estimated at 20.06 in the intervention group and 16.27 in the control group (rate ratio, 1.23; 95% CI, 0.78-1.94; *P* = .36) (Table 4). Results of per-protocol and exploratory analyses are given in eAppendix 9 and eAppendix 10 in Supplement 2.

## Discussion

Our findings do not indicate that systematic dementia screening of NH residents through MDTM resulted in a lower rate of ED transfer. To our knowledge, this is the first large-scale randomized clinical trial to test the effectiveness of international recommendations for the diagnosis of Alzheimer disease in NHs.^1^ The high rate of new diagnoses of dementia (approximately 41%) among residents who were not previously formally diagnosed in the intervention group confirms the underawareness of dementia in NHs.^2^ However, in spite of these new diagnoses of dementia and the concomitant recognition of various associated geriatric syndromes such as malnutrition, risk of falls, depression, or behavioral disturbances (Table 3) during the MDTM in the intervention group, the overall rate of ED transfers was not lower than in the control group during the first 12 months of follow-up.

Rates of transfer of NH residents to the hospital vary between countries but are approximately 40% per year.^11,12^ Transfers are mainly to the ED, with a high rate of ED transfer for older people with dementia.^4,13,14^ Underrecognition of dementia can result in poor understanding of a resident’s behavior and inappropriate therapy, ultimately leading to an inappropriate care plan. When residents with dementia are hospitalized, they are generally exposed to iatrogenic events and delirium^15^ and have a longer hospital stay and greater functional decline than elderly people with similar health conditions but without dementia.^1,12,16^ On the other hand, previous research has indicated that dementia plays a moderating role in the associations between acute diseases and ED transfers.^17^ In our study, the mean proportion of participants with at least 1 ED transfer during the first 12 months of follow-up in the overall population was high (around 14%). It was higher, but not significantly so, in the intervention group than in the control group, but ED transfer rate varied greatly between NHs (Table 4; eTable 2 in Supplement 2), suggesting large disparity of practices among NHs. The intervention was mainly focused on dementia screening rather than specifically on reducing ED transfers (or the prevention of acute health conditions) and was probably too weak to set a downward trend in the routine practice of transfers and the habits of each NH staff.

These results must be analyzed with caution. A reduced ED transfer rate was expected in residents newly diagnosed with dementia (approximately 41% of the intervention group diagnosed with highly probable or suspected dementia), but our intervention may have also resulted in an increased ED transfer rate among residents newly confirmed without dementia (54% of the intervention group) (Table 3). Awareness that a resident does not have dementia may in fact change the attitude of the NH staff and encourage a maximal approach to care. We speculate that our neutral results may be explained by a lower hospitalization rate of residents with a highly probable or suspected diagnosis of dementia, balanced by a higher hospitalization rate of residents with confirmed exclusion of diagnosis of dementia. This hypothesis may explain our counterintuitive results and support better quality of care. However, the supplementary analyses tend to refute this hypothesis (eTable 3 in Supplement 2). In fact, subgroup analysis according to dementia status found no statistically significant differences in ED transfer rate (16.9% of residents diagnosed with dementia at the first MDTM vs 13.6% with no diagnosis of dementia).

We can also speculate that our neutral results may be explained by the lack of compliance to the care plan and recommendations proposed by the MDTMs by the residents’ GPs. However, subgroup analyses found no statistically significant differences in ED transfer rate comparing the residents whose GP followed the recommendations of the MDTMs vs control (eTable 4 and eTable 5 in Supplement 2). Similar results were found when we compared the ED transfer rate between GP compliant residents vs GP noncompliant residents within the intervention group (eTable 6 and eTable 7 in Supplement 2). In our study, we did not collect data on the compliance of the NH staff to the care plan and recommendations proposed by the MDTMs.

Our study also suggests that the probability of at least 1 inappropriate hospitalization was significantly higher in the intervention group than in the control group (Table 4). These results suggest that the dementia screening process in the intervention group did not influence the probability of ED transfer, whatever the final diagnosis and the care plan proposed during the MDTM. The reason why residents in the intervention group were statistically more often transferred inappropriately to the ED (whatever the final diagnosis of dementia) remains unclear, but supports the suggestion that too much medication may be harmful in this population.^18^ However, this finding must be interpreted with caution because it consisted of an exploratory analysis performed in a non–randomly selected subgroup of participants and was restricted by data availability (only participants whose hospitalization report was available were included in this analysis).

Current evidence and expert opinion indicate that person-centered care and dementia care mapping in patients with Alzheimer disease improve the management of symptoms such as behavioral disturbances as well as clinical outcomes such as NH admission.^1^ However, these findings were made in community-dwelling patients^19^ rather than NH residents with severe dementia or disability. In this specific NH population, this approach may be less beneficial or even pointless. There is growing concern in the scientific community about overdiagnosis or screening programs that diagnose diseases that would not cause harm to the patient. One could argue that the residents with dementia were not previously diagnosed because no clinically significant problem arose from their disease. Possibly, after systematic screening, diagnosis of dementia may have resulted in overuse of pharmaceutical agents that endangered the residents’ health rather than enhancing it, and in performance of brain imaging or blood tests that raised the risk of new diagnoses and new investigations, thus distressing the resident, their relatives, and the NH staff; incurring unnecessary expense; and increasing the rate of inappropriate ED transfer. This may have occurred in particular when NH staff were not trained in dealing with people with dementia (as suggested by our exploratory results in NHs without a special Alzheimer disease care unit) (Table 4) and it may also have diverted the already stretched human resources of the NH to nonuseful tasks.

### Limitations

To our knowledge, the IDEM study is the largest randomized clinical trial to examine the benefit of systematic dementia screening in older people. However, several limitations of the study should be noted. First, IDEM was a national multicenter study in which NHs took part on a voluntary basis. They were not representative of all NHs. However, it seems unlikely that the same intervention in nonvolunteering NHs would have resulted in better outcome. Second, owing to the constraints of the study, its long duration, and NH staff turnover, 25 NHs withdrew their participation during the first 3 months (preselection period), resulting in an underpowered sample size. The initial objective was to include 2000 residents to have 80% power to detect a 30% reduction assumption of ED transfer rate in the intervention group. Whether higher recruitment would have resulted in a significantly lower rate of ED transfers in the interventional group remains unknown. However, the observed trend of higher ED transfer in the intervention group makes this hypothesis unlikely.

We did not perform any interim analysis for this nonpharmacologic treatment intervention, as interim analysis was not planned at the beginning of the trial. Given the insufficient number of participants included, a 12-month interim analysis could have been relevant; however, owing to the expected insufficient number of participants, we opted for a longer period of exposure to the intervention (adding a second MDTM) and a longer-term (18-month) outcome measure.

The data for staff to resident ratio for each NH were not collected in the study. It would have been interesting to describe the exact typology of the staff in the different NHs. However, in France we have indicators of care load (GMP, an NH level-of-dependence score; and PMP, an NH comorbidity score), which are proxies defining the staffing requirements of the different structures. In this study, we collected GMP. Despite a comparable GMP in both groups at baseline (Table 1), we cannot exclude a disparity in the distribution of the different trades between the 2 groups.

## Conclusions

In conclusion, this study does not preclude benefits from the diagnosis of dementia currently made in residents before or after NH admission. However, our results do not support the recommendations for systematic screening of all cases of dementia in NH.

## Notes

Supplement 1. **Trial Protocol**

Supplement 2. **eAppendix 1.** Role of Coordinating Physician in NH **eAppendix 2.** Stratification Criteria of Cluster Randomization **eAppendix 3.** GIR Score **eAppendix 4.** Inclusion and Visits in NHs **eAppendix 5.** Content of MDTM **eAppendix 6.** Classification of Hospitalizations as Appropriate or Inappropriate **eAppendix 7.** Per-protocol Analyses **eAppendix 8.** Exploratory Analyses **eAppendix 9.** Results of Per-protocol Analyses **eAppendix 10.** Results of Exploratory Analyses **eReferences.** **eFigure.** Study Flow-chart **eTable 1.** Comprehensive Geriatric Assessment at Inclusion and End of Study Visits (mITT Population) **eTable 2.** Assessment of Intervention Effect on Primary and Secondary Endpoints Observed in the 64 Nursing Homes in Person-Years (mITT Population, N=1401) **eTable 3.** Estimation and Test of Effect of Dementia Diagnosis on Risk of ED Transfer During 12-Month Follow-up (mITT Population) (N=1401) **eTable 4.** Assessment of Effect of Following the Recommendations of the First MDTM by GP on Risk of ED Transfer During 12-Month and 18-Month Follow-up (Subjects With MDTM1 Recommendations Followed by GPs Versus Control) (N=1086) **eTable 5.** Assessment of Effect of Following the Recommendations of the First and the Second MDTMs by GP on Risk of ED Transfer During 18-Month Follow-up (Subjects With Two MDTMs Recommendations Followed by GPs Versus Control) (N=960) **eTable 6.** Assessment of Effect of Following the Recommendations of the First MDTM by GP on Risk of ED Transfer During 12-Month and 18-Month Follow-up (Subjects With MDTM1 Recommendations Followed by GPs Versus Those With MDTM1 Recommendations Not Followed by GPs) (N=445) **eTable 7.** Assessment of Effect of Following the Recommendations of the First and the Second MDTMs by GP on Risk of ED Transfer During 18-Month Follow-up (Subjects With Two MDTMs Recommendations Followed by GPs Versus Those With Two MDTMs Recommendations Not Followed by GPs) (N=371)

Supplement 3. **Data Sharing Statement**
