# Supplementary material for: Systematic Dementia Screening by Multidisciplinary Team Meetings in Nursing Homes for Reducing Emergency Department Transfers: The IDEM Cluster Randomized Clinical Trial
Source: JAMA Netw Open. 2020 Feb 26;3(2):e200049. doi: 10.1001/jamanetworkopen.2020.0049 (PMC7137681; doi:10.1001/jamanetworkopen.2020.0049)
Supplement: Supplement 1. — Trial Protocol [file jamanetwopen-3-e200049-s001.pdf]

Promoter:
CHU de Toulouse, Hôtel-Dieu, 2 rue Viguerie, 31052 Toulouse cedex 9

Coordinator investigator:
Professor Yves Rolland, Gérontopôle de Toulouse, La Grave Hospital, Cité de la Santé, 1 Place Lange, 31059 Toulouse cedex 9
rolland.y@chu-toulouse.fr

SUMMARY

Interest of dementia tracking in nursing home: contribution of multidisciplinary team meeting (MDTM) in Alzheimer's disease and related diseases- IDEM Study

JUSTIFICATION

Access to the diagnosis of Alzheimer's disease and other dementias remains a problem in France especially after the age of 80 (Expertise INSERM 2007). Epidemiological data currently available in France report that in the general population, only half of patients with Alzheimer's disease are diagnosed (Gallez C., 2005).
The absence of a diagnosis of dementia at an early stage reduces the chances of optimal patient management. A consequence of the non-diagnosis of dementia is the inappropriate use of care services, including increased recourse to emergency hospitalization (Gordon et al., 1991; Kayser-Jones et al., 1989; Kerr et al., 1991).

Although the value of screening in the general population is not conceivable in the absence of curative treatment of dementia, the question of systematic detection of dementia cases arises in nursing homes (NH) because of the high frequency of dementia (50 to 70% depending on the case) and its under-diagnosis. While, the benefit of appropriate care is recognized even at an advanced stage of the disease (Expertise INSERM 2007), no study to our knowledge has been conducted on the interest of a systematic detection of dementia in nursing homes.

OBJECTIVES

The objective of the IDEM study is to evaluate the impact of a systematic screening of cases of dementia performed in the framework of MDTM (Multidisciplinary Team Meeting) in nursing home (NH).

The interest of this approach is evaluated on the rate of emergency department (ED) transfers at one year. Our hypothesis is that a systematic identification of dementia cases in EHPAD reduces the rate of ED transfers.

The secondary objectives are to determine the impact of systematic screening of dementia on the quantity and type of drug prescriptions (neuroleptics, benzodiazepines, antidepressants, hypnotics, anxiolytics, thymo-regulators, anti-cholinesterases, memantine) and non-drug treatments (physiotherapy, occupational therapy, psychological counseling, dietetics, speech therapy), the appropriateness or inappropriateness of hospitalizations (including emergencies), the prevalence of psycho-behavioral disorders, the residents' process of entry into the dependency, the quality of life of residents, the burden of the NH staff, the advanced care planning measures (advance directives ...) and the overall cost of care.

Diagram of the study

Multicenter, randomized by NH (1:1) comparing two-parallel groups:

- an intervention group, corresponding to NH that set up MDTM to identify patients with dementia and to discuss an appropriate care plan,
- and a control group, corresponding to NH that continued their usual practice.

Inclusion criteria

During the inclusion period, all the residents of participating NH who meet the study criteria are included in the study: subjects aged 60 years or older, without diagnosed or documented dementia, not bedridden [Groupe Iso-Ressource (GIR) >1), living in the NH for at least one month at inclusion, with a life expectancy of more than one year and without any disease likely to jeopardize his/her participation in the study.

Research processes

The project is based on the REHPA network (Research in NH), set up in 2008 by our team and regrouping 240 NH with the aim of setting up clinical research projects specifically adapted to NH population. The cluster randomization takes into account some characteristics of NH related to the type of institution and the type of residents.

After being trained, the coordinating physicians practicing in the NH of the intervention group carry out a comprehensive geriatric assessment for each participating resident. The results of the various

tests are discussed between the coordinating physician and an expert from a nearby “memory resource and research center (MRRC)” or a “memory clinic (MC)” during a MDTM on Alzheimer's disease and related diseases.

Information on hospitalizations, drug and non-drug prescriptions, the appropriateness of hospitalization, the prevalence of psycho-behavioral disorders, residents’ dependence and quality of life, the burden of the NH staff, the planning of advance directives and the overall cost of care are collected during follow-up. These monitoring data are collected by an ARC via a secure website (REHPA web page), or if necessary by mail or phone calls. The overall cost of care is provided by the French National Health Insurance Fund.

Calculation of the number of subjects

The number of subjects needed to meet our main objective was calculated by hypothesizing a bilateral test with an alpha risk of 5% and a beta risk of 20% (80% power). Based on previous data, we estimated the incidence of ED admissions of 24% at 12 months in the control group. To detect a 30% reduction of the rate of transfers to ED in the intervention group, with a two-tailed test and an α risk of 5% and 80% power, 1000 subjects were required in each group, taking into account an intracluster correlation coefficient (ICC) at 0.02, 20% of attrition rate and the inflation factor related to contamination between groups of 5 to 10%, during 12-month trial.

Inclusion and patients’ follow-up

After a 3-month preselection period to identify the NH residents meeting the study criteria, the preselected residents in both groups have an inclusion visit in the NH realized by the coordinating physician. Each NH include all of its residents in a time interval of two months. Sociodemographic and medical data are collected in both groups. The residents in the intervention group also undergo a comprehensive geriatric assessment. The included residents are followed for 18 months. At the end of follow-up, all the residents in both groups undergo a final visit in the NH performed by the coordinating physician including a simplified comprehensive geriatric assessment.

Expected outcomes

The identification of residents with dementia in NH appears to be a relevant means of optimizing the care of the resident and, in particular, reducing the need for transfers to the ED. The opinion given by a MDTM on Alzheimer's disease and related diseases (plan of care, medication, non-pharmacological

approaches, advances directives…) would prevent certain frequent situations during dementia leading to hospitalizations.

The demonstration of the interest of a MDTM in the systematic detection of dementia in NH could lead to a generalization of this type of procedure at the national and international level.

REFERENCES

- Expertise collective sur la Maladie d'Alzheimer ; Enjeux scientifiques, médicaux et sociétaux. Les Editions Inserm 2007, 101 rue de Tolbiac, 75013 Paris.
- Gallez C. Rapport sur la maladie d'Alzheimer et maladies apparentées. Office Parlementaire d'Evaluation des Politiques de Santé (OPEPS), 2005.
- Gordon M, Klapecki KC, Wilson DB. Emergency care and the patient in the long-term care facility. CMAJ. 1991 Jul 1;145(1):19-21.
- Kayser-Jones JS, Wiener CL, Barbaccia JC. Factors contributing to the hospitalization of nursing home residents. Gerontologist. 1989 Aug;29(4):502-10.
- Kerr HD, Byrd JC. Nursing home patients transferred by ambulance to a VA emergency department. J Am Geriatr Soc. 1991 Feb;39(2):132-6.

Interest of dementia tracking in nursing home: contribution of multidisciplinary team meeting (MDTM) in Alzheimer's disease and related diseases- IDEM Study

- IDEM Study-

Code : 0910701

Statistical Analysis Plan (SAP)

VERSION : 1.0

Date : 07/22/2016

N° of National Agency for the Safety of Medicines and Health Products : 2009-A01062-55

This study was supported by a grant from the French Ministry of Health

(Clinical Research Hospital Program, PHRC 2009).

Biostatistician author of the SAP:
Elodie Tournay, CHU de Toulouse, Unité de Soutien Méthodologique à la Recherche (USMR)
Faculté de Médecine, 37 allées Jules Guesde, 31062 Toulouse cedex 9
Tel : 05 61 14 56 73 / e.mail : tournay.e@chu-toulouse.fr

Methodologist:
Sandrine Andrieu, CHU de Toulouse, Unité de Soutien Méthodologique à la Recherche (USMR)
Faculté de Médecine, 37 allées Jules Guesde, 31062 Toulouse cedex 9
Tel : 05 61 14 59 32 / e.mail : sandrine.andrieu@univ-tlse3.fr

Study promoter:
CHU de Toulouse, Hôtel-Dieu, 2 rue Viguerie, 31052 Toulouse cedex 9

Coordinator investigator (multicentre study):
Professor Yves Rolland, Gérontopôle de Toulouse, La Grave Hospital, Cité de la Santé, 1 Place Lange, 31059 Toulouse cedex 9
Tel : 05 61 77 70 21 / Fax : 05 61 77 64 75 / e.mail : rolland.y@chu-toulouse.fr

HISTORY OF THE DOCUMENT

VERSION	DATE	DESCRIPTION DES MODIFICATIONS
V1.0	07/22/2016	First validated version

Contents

1 PURPOSE OF THE STATISTICAL ANALYSIS PLAN 8
2 CHANGES IN RELATION TO THE PROTOCOL 8
3 SUMMARY OF THE PROTOCOL 8
3.1 MAIN OBJECTIVE 8
3.2 SECONDARY OBJECTIVES 9
3.3 CALCULATION OF THE NUMBER OF SUBJECTS 9
4 EVALUATION CRITERIA 10
4.1 PRIMARY ENDPOINT 10
4.2 SECONDARY ENDPOINTS 10
4.3 ADDITIONAL ENDPOINTS 12
5 STUDY POPULATIONS 13
5.1 INTENTION TO TREAT POPULATION (ITT) 13
5.2 FIRST PER PROTOCOL POPULATION (PP1) 13
5.3 SECOND PER PROTOCOL POPULATION (PP2) 13
6 STATISTICAL ANALYZES 13
6.1 PRINCIPAL ANALYSIS OF THE PRIMARY ENDPOINT 13
6.2 COMPLEMENTARY ANALYZES OF THE PRIMARY ENDPOINT 14
6.3 ANALYSIS OF THE SECONDARY ENDPOINTS 15
6.3.1 General 15
6.3.2 ED transfer during 18 months 16
6.3.3 The quantity and type of non-drug treatments 17
6.3.4 The appropriateness or inappropriateness of hospitalizations 17
6.3.5 The prevalence of psycho-behavioral disorders 17
6.3.6 The residents' process of entry into the dependency 17
6.3.7 The residents' quality of life 18
6.3.8 The burden of the NH staff 18
6.3.9 The advanced care planning measures (advance directives ...) 18
6.4 ANALYSIS OF THE ADDITIONAL ENDPOINTS 18
6.5 DESCRIPTIVE ANALYZES 19
6.5.1 General 19
6.5.2 Description of subjects not included 19
6.5.3 Description of data collected at inclusion 19
6.5.4 Description of the data collected during the first multidisciplinary team meeting (MDTM1) 20
6.5.5 Description of the data collected during the follow-up 21

6.5.6 Description of the data collected at the end-of-study visit 21
6.5.7 Study dropout 22
6.6 SENSITIVITY ANALYZES 22
6.7 ANALYSIS IN SUBGROUPS 22
7 MISSING DATA MANAGEMENT 22
8 STATISTICAL SOFTWARE USED 23

1 PURPOSE OF THE STATISTICAL ANALYSIS PLAN

This document describes the statistical methodology that will be used to meet the objectives of the study as well as all the analyzes that will be performed and methods of deriving variables.

The analysis of the impact of the intervention on the overall cost of patient care (secondary objective) will be carried out separately by a medico-economic service and will not be described in this SAP.

The analysis of the impact of the intervention on the quantity and type of drug prescriptions (secondary objective) will be the subject of a thesis by a pharmacy student and will therefore not be described in this SAP.

The statistical analysis will begin after the validation of this analysis plan by the scientific council of the study and reception of the cleaned and frozen database.

2 CHANGES IN RELATION TO THE PROTOCOL

Contrary to what is stated in the protocol, the statistical analyzes will not be performed blind. This is not possible given the differences between the data collected in the 2 groups (i.e. geriatric assessment and multidisciplinary team meetings (MDTM) performed only in the intervention group).

3 SUMMARY OF THE PROTOCOL

3.1 Main Objective

The objective of our project is to evaluate the impact of a systematic detection of dementia cases carried out with residents of Nursing Homes (NH) during a MDTM on the use of the care system and more specifically on the admission rate to emergency department (ED). Like MDTM in oncology, this MDTM on Alzheimer's disease and related diseases aims to discuss the diagnosis of dementia and the health project of NH residents according to a simplified Comprehensive Geriatric Assessment (CGA) carried out by the coordinating physician.

3.2 Secondary Objectives

The secondary objectives of this study are to determine the impact of systematic detection of dementia during MDTM on:

a. the quantity and type of drug prescriptions (neuroleptics, benzodiazepines, antidepressants, hypnotics, anxiolytics, thymo-regulators, anti-cholinesterases, memantine) and non-drug treatments (physiotherapy, occupational therapy, psychological counseling, dietetics, speech therapy),
b. the appropriateness or inappropriateness of hospitalizations (including emergencies),
c. the prevalence of psycho-behavioral disorders,
d. the residents' process of entry into the dependency,
e. the residents' quality of life,
f. the burden of the NH staff
g. the advanced care planning measures (advance directives ...)
h. and the overall cost of care.

3.3 Calculation of the number of subjects

The number of subjects required to meet our main objective was calculated by assuming a bilateral test with an alpha risk of 5% and a beta risk of 20% (80% power). On the basis of REHPA survey data (Rolland Y et al. 2009), 13.5% of all residents reported in the REHPA survey were admitted to hospital services in 3 months, an estimate of 54% in 1 year. By limiting the population of the REHPA survey to subjects with the inclusion criteria defined in IDEM, 12% were admitted to hospital services in 3 months, an estimate of 48% at 1 year. According to the same survey, of the 48% of all hospitalizations, half were via ED. Indeed, we can estimate an incidence of ED transfers of 24% at 1 year in the non-intervention group. Assuming a 30% reduction in the ED transfer rate in the intervention group, the 12-month ED transfer rate will be 16.8%. 516 subjects are needed in groups, taking into account the cluster effect with an ICC set at 0.02 of an attrition rate of 20% (average known % of deaths per year from REHPA data), the minimum number of subjects per group varies from 950 to 1050 depending on the inflation factor linked to the contamination between the groups, which has been varied from 5 to 10%.

In total, 1,000 residents per group or 2,000 residents must be included. Taking into account an inclusion number of 30 residents on average per NH, 35 institutions per arm are necessary. Given the potential of 240 NH of REHPA network, this number of institution is realistic to achieve. In total 89 nursing homes have volunteered.

4 EVALUATION CRITERIA

4.1 Primary endpoint

The primary endpoint of this study, collected by subject, will be the occurrence of at least one admission to a hospital ED during a one-year follow-up period (i.e., yes / no binary criterion).

4.2 Secondary endpoints

To complete the main analysis of the impact of MDTM on transfers to ED, the following additional criteria will also be analyzed: the occurrence of at least one admission to a hospital emergency department during an 18-month follow-up period (i.e. binary yes / no criterion). All of the following secondary endpoints are defined according to the secondary objectives to which they respond.

To determine the impact of systematic detection of dementia during MDTM on:

a- The quantity and type of non-drug treatments

The endpoint to meet this objective will be the changes of non-drug (paramedical) prescriptions such as physiotherapy, occupational therapy, psychomotricity, psychological counseling, dietetics, speech therapy or other care during the 18 months of follow-up since the inclusion (ie binary yes / no criterion). The changes will be analyzed first globally then separately for each type of care cited. The changes include stopping, increasing or decreasing the periodicity and the introduction of a care.

b- The appropriateness or inappropriateness of hospitalizations (including emergencies)

The endpoint will be the occurrence of at least one admission judged by experts as "not appropriate" or as "avoidable" in a hospital service (including emergencies) during the 18 months of follow-up (i.e. binary yes / no criterion).

Note: Only hospitalizations for which a report was available were classified by the experts.

c- The prevalence of psycho-behavioral disorders

The endpoint will be the total score obtained in the Neuropsychiatric Inventory caregiver version (NPI) (Cummings et al., 1994, Robert et al., 1998) at the end-of-study visit (i.e., quantitative criterion).

d- The residents' process of entry into the dependency

The endpoints will be:

- the difference between the GIR scores obtained between the end-of-study visit and the inclusion visit (i.e. quantitative criterion)
- the total score obtained on the 4 items of Lawton's IADL (Lawton et al., 1969) at the end-of-study visit. This score varies between 0 and 4. (i.e. quantitative criterion).

e- The residents' quality of life

The endpoint will be the total score obtained by the QOL-AD scale (Ready et al., 2007) at the end-of-study visit (i.e. quantitative criterion).

f- The burden of the NH staff

This endpoint is collected at the level of NH centers and not at the subject level. The hardness task of caregivers will be appreciated from:

- the total number of work interruptions recorded during the 18 months of follow-up in each NH center relative to the number of beds / residents in the NH (i.e. quantitative criterion)
- the number of cumulative days of work interruptions recorded during the 18 months of follow-up in each NH center relative to the number of beds / residents in the NH (i.e. quantitative criterion)

Note 1: Only the 18-month period following the first wave of inclusion will be considered here because not all centers have achieved a second wave of inclusion.

Note 2: The number of beds / residents in each NH will be used as a measure of the number of caregivers working in each NH. Indeed, the number of NH caregivers is counted according to the number of residents / beds. Thus, the number of work interruptions in each NH will be transformed into the number of interruptions per 100 beds / residents in order to be compared between the centers.

Note 3: The turnover and the departures of the caregivers, were not collected.

g- The advanced care planning measures (advance directives ...)

The endpoint will be the addition of at least one advance directive, whatever the type, in the resident's file during the 18 months of follow-up. The additions will be analyzed first globally then separately for each type of advance directive defined below.

Here are the 10 items collected in the CRF for the two intervention and control groups concerning "The planning of specific therapeutic measures" (advance directives):

- Prescriptions of therapeutic drug protocols for the management of behavioral disorders
- Requirements for non-drug therapeutic protocols for the management of behavioral disorders
- Prescriptions of therapeutic drug protocols for the management of pain
- Prescriptions of therapeutic drug protocols for the management of end-of-life discomfort symptoms (other than pain)
- Requirements for non-medical therapeutic protocols for the management of end-of-life discomfort symptoms (other than pain)
- No transfer in intensive care
- No transfer to ED
- No resuscitation
- No hospitalization
- Other

4.3 Additional endpoints

The following additional criteria for judgment have been added:

- the occurrence of at least one admission (planned or via ED) in any hospital service during the 18-month follow-up period (i.e. binary yes / no criterion).
- the occurrence of at least one planned admission to any hospital service except ED during the18-month follow-up period (i.e., yes / no binary criterion).
- the occurrence of at least one emergency admission in an out-of-emergency hospital service during the 18-month follow-up period (i.e. binary yes / no criterion).

5 STUDY POPULATIONS

5.1 Intention to treat population (ITT)

The ITT population will consist of all subjects included in the study who have at least 1 month of follow-up. That is, all residents of the randomized NH centers who performed the inclusion visit and who did not leave the study within one month of the start of the follow-up (i.e. the end of the inclusion period in the center).

5.2 First Per Protocol population (PP1)

The population PP1 will be composed of subjects of the ITT population for whom:

- all the criteria for inclusion and exclusion are respected (not included wrongly);
- the intervention was carried out for the subjects of the intervention group (subject's file discussed during the first MDTM);
-

5.3 Second Per Protocol population (PP2)

The population PP2 will be composed of subjects of the ITT population for whom:

- all the criteria for inclusion and exclusion are respected (not included wrongly);
- the 2 interventions were carried out for the subjects of the intervention group (subject's file discussed during the 1st and 2nd MDTM);

6 STATISTICAL ANALYZES

6.1 Principal analysis of the primary endpoint

The number and percentage of subjects who had at least one admission to a hospital ED in a one-year follow-up period will first be described by randomization group of the patient's NH (intervention or control) and in total.

The 1-year follow-up period for each subject begins at the end of the wave of inclusion in the center (i.e. start date of hospitalization collection).

The primary analysis of the effectiveness of management will compare the ED transfer rate between the two groups after a 1-year follow-up. To account for intra-center correlation (i.e., non-independence between observations of the same center) the effect of the intervention will be tested using a logistic regression model with random effects. The center randomization group (i.e. NH) subjects will be considered as a fixed effect and the center as a random effect

to model the risk of a transfer to the ED. The risk of the first species for this main analysis is set at 5% (confirmatory test).

The intra-class correlation coefficient will also be calculated from this model.

Population of analysis: The main analysis of the primary efficacy endpoint of the intervention will be carried out on the subjects of the ITT population. Thus, subjects who left the study before 1 year of follow-up will still be considered in this analysis if their follow-up is at least 1 month. For example, a patient who died after 4 months of follow-up without ED transfer will have a primary endpoint equal to "No". This will lead to a possible underestimation of the rates of subjects admitted to ED in 1 year in the 2 groups but should not bias the comparison between the groups since the follow-up times should be close between the 2 groups.

As sensitivity analyzes, this analysis will also be performed on the PP1 population in addition to the ITT analysis.

6.2 Complementary analyzes of the primary endpoint

First, a second mixed logistic regression model will be used to study the effect of the intervention on the risk of admission to the ED in the first year, taking into account potential confounders of age, co-morbidities (Charlson), dependency (GIR score), antecedents of psychiatric pathologies and the presence of cognitive decline at inclusion. The effect of the intervention will be tested with a risk of 5% (exploratory test).

Then, in order to consider the differences in the duration of follow-up caused by the deaths and the anticipated stopping of study, other complementary analyzes will be conducted on the primary endpoint.

- Incidence rates defined as the number of emergency room admissions per 100 person-years (i.e. 100 persons followed exactly 1 year in NH) will be calculated for each NH and a statistical test will be performed to determine if the difference between the incidence rates in both groups is significant (exploratory test at 5%). To calculate incidence rates, the at-risk follow-up time for each subject will first be calculated as the time in years between the start date of follow-up (i.e. end date of inclusions in the center) and the date of point (i.e. start date of follow-up plus 1 year) or the date of premature dropout of the study for the subjects who stopped the study before 1 year of follow-up MINUS possible durations of hospitalization (i.e. the subjects are no longer at risk of transfer to the ED

when they are hospitalized). Then, for each subject, the number of transfer to ED that occurred during the risk period will be counted. The incidence rate for each NH will be calculated as the total number of ED transfers divided by the sum of the at-risk follow-up times for the center's subjects and will be transformed per 100 person-years.

- Finally, a survival analysis will be carried out to study the delays until the occurrence of the event (i.e. first transfer to the ED). In this study, death is a competitive risk to consider in the analysis given that the risk of having an event after death is zero (i.e., no independence between the two risks). Standard Kaplan-Meier survival analyzes do not lead to valid results for a specific risk if failures for another cause are treated as censorship. A suitable method is the use of cumulative impact functions that represent the cumulative probability of failure due to a specific cause over time. Cumulative incidence functions will be calculated for each group considering the death as a competitive risk and the difference between the functions of the 2 groups and the 5% Alpha risk method will be tested (exploratory test). Cumulative incidence rates estimated at 1 year will be presented for each group with their 95% confidence interval. Deadlines will be calculated in days between the start date of follow-up and:
  - the date of first ED transfer for the subjects having had the event studied in the year or
  - the date of death for subjects who died in the year (subjects with competitive risk) or
  - the premature dropout date of the study if dropped out for a reason other than death before one year (censored subjects) or
  - the point date for the remaining subjects who will also be censored (i.e. end date of inclusions in the center plus 1 year)

Population of analysis: All complementary analyzes of the primary endpoint will be performed on the ITT population. The analysis of incidence rates will also be performed on the PP1 population.

6.3 Analysis of the secondary endpoints

6.3.1 General

All secondary endpoints will be previously described by NH randomization group of subjects (intervention or control) and in total. The quantitative criteria will be summarized by their

mean, standard deviation, median, 1st and 3rd quartile (Q1 and Q3), minimum and maximum. For the qualitative criteria, the number and percentage corresponding to each category will be presented.

All statistical tests performed to meet the secondary objectives will be bilateral with a risk of the first species at 5%. All these tests will have to be considered as purely exploratory not guaranteeing a global risk on the study of 5% (multiplicity of the tests).

The 18-month follow-up period for the secondary endpoints begins at the end of the inclusion wave in the center (i.e., the start date for the collection of judgment criteria).

Population of analysis: The analyzes on the secondary criteria of judgment will be made on the subjects of the ITT population for which the endpoint is available, except if another population is defined explicitly.

6.3.2 ED transfer during 18 months

The first admission to an ED within 18 months of the start of the follow-up will be the event considered.

The main analysis (logistic regression with random effects) and the additional analyzes planned on the main judgment criterion will be carried out again considering this time 18 months of follow-up for each subject.

To calculate incidence rates, the risk follow-up time for each subject will be the time in years between the start date of follow-up (i.e. end date of inclusions in the center) and the date of point (i.e. start date follow-up plus 18 months) or the premature dropout date of the study for subjects who stopped the study before 18 months of follow-up MINUS possible hospitalization times.

For the survival analysis, the time will be calculated in days between the start date of follow-up and:

- the date of first hospitalization in the emergency department for subjects having had the event studied within 18 months or
- the date of death for the subjects who died within 18 months without having gone to the ED beforehand (subject with competitive risk) or
- the premature dropout date of the study if dropped out for any reason other than death within 18 months (censored subjects) or

- the date of the point for the remaining subjects who will also be censored (i.e. end date of the inclusions in the center plus 18 months)

Population of analysis: The main analysis will be carried out on the population ITT as well as on the populations PP1 and PP2. The additional analyzes will be done on the ITT, the analysis of the incidence rates will also be carried out on the PP1 and PP2 populations.

6.3.3 The quantity and type of non-drug treatments

The modification of the prescription of a non-drug treatment will be modeled using a mixed logistic regression model with the randomization group as the fixed effect and the center as a random effect to estimate the impact of the group on the occurrence of the criterion.

6.3.4 The appropriateness or inappropriateness of hospitalizations

The analysis will compare the risk of "inappropriate" or "avoidable" admission to a hospital ward (including emergencies) within 18 months of follow-up between the two groups using a mixed logistic regression model. The randomization group will be considered a fixed effect and the center as a random effect.

6.3.5 The prevalence of psycho-behavioral disorders

The total score obtained in the NPI version for the nursing team at the end-of-study visit will be compared between the 2 groups using a mixed model of analysis of variance (comparison of mean values). The randomization group will be considered as a fixed effect and the center as a random effect.

6.3.6 The residents' process of entry into the dependency

The difference between the GIR scores at the end-of-study visit and during the inclusion visit will be compared between the 2 groups using a mixed model of covariance analysis (comparison of mean values by adjusting for the inclusion score). The randomization group and the inclusion score will be considered as fixed effects and the center as a random effect. The total score obtained on the 4 items of the Lawton IADL at the end-of-study visit will be compared between the 2 groups using a mixed model of analysis of variance (comparison of

mean values). The randomization group will be considered as a fixed effect and the center as a random effect.

6.3.7 The residents' quality of life

The total score obtained by the QOL-AD scale at the end-of-study visit will be compared between the 2 groups using a mixed model of analysis of variance (comparison of mean values). The randomization group will be considered as a fixed effect and the center as a random effect.

6.3.8 The burden of the NH staff

This analysis will be done at the center level and not at the subject level. The total number of work interruptions and the number of cumulative days reported per 100 residents registered during the study duration in each EHPAD center will be compared between the 2 groups using a Student's test to compare the mean values (Two- Sample T-test).
Population of analysis: These analyzes will be made **on the population of randomized NHs.**

6.3.9 The advanced care planning measures (advance directives ...)

The presence of prescriptions in the residents' file at the end of the study will be compared between the 2 groups using a mixed model of logistic regression. The randomization group will be considered as a fixed effect and the center as a random effect.

6.4 Analysis of the additional endpoints

A mixed logistic regression model will be conducted to study the effect of the intervention on the risk of admission to a hospital ward. The effect of the intervention will be tested with a risk of 5% (exploratory test). The incidence rates of hospitalizations over a period of 18 months of follow-up will be calculated in each group.
These analyzes will be carried out **on ITT, PP1 and PP2 populations.**

6.5 Descriptive analyzes

6.5.1 General

Additional descriptive analyzes will be produced on the inclusion data as well as data collected during the study that are not the endpoints. The quantitative variables will be summarized by their mean, standard deviation, median, 1st and 3rd quartile (Q1 and Q3), minimum and maximum. For qualitative variables, the number and percentage corresponding to each category will be presented.

Descriptions will be performed by randomization group of the subjects' NH and in total.

The differences between the 2 groups will not be tested statistically in these descriptive analyzes.

Population of analysis: The descriptive analyzes will be done **on the subjects of the ITT population for which the data is available**, except if another population is defined explicitly.

6.5.2 Description of subjects not included

The number of residents of the randomized NH not included in the study will be described as well as the reasons for not including.

6.5.3 Description of data collected at inclusion

The characteristics found in the randomized NH centers (randomization stratification factors, number of residents included) will first be described.

The following characteristics of the subjects identified for inclusion in the study will be described for the entire analysis population and will be described in the 2 groups (intervention, control):

- Criteria for inclusion and non-inclusion;
- Sociodemographic characteristics (age, sex, marital status, education, mother tongue (French yes / no), occupation (yes / no), seniority in the NH (in months);
- Medical history and associated pathologies (visual or auditory impairment, smoking and alcohol consumption, total Charlson Comorbidity Index score);

- Cognitive antecedents (progressive decline yes / no, MMSE test yes / no and score, cognitive assessments yes / no and type of assessment);
- Family history of dementia (presence of family history of dementia yes / no);
- Hospitalizations during the last 3 months by type of hospitalization (planned or in emergency) and by type of service;
- Ongoing drug and non-drug treatments at inclusion by type of treatment;
- Anticipated prescriptions by type;
- GIR total score at inclusion.

The following results from the Comprehensive Geriatric Assessment (CGA) obtained during the inclusion visit only for the intervention group, will be described:

- Overall MMSE score;
- Results of the clock test (score and normality);
- Results of the Borson Mini-Cog (normality);
- Overall score of the 5 word test;
- Categorical verbal fluency test results (total words generated, total acceptable words);
- Confusion Assessment Method (CAM) test result (acute confusional state yes / no);
- Results of the Mini-Nutritional Assessment (MNA) test (total score, assessment);
- Result of the one-leg standing balance test (normality);
- Neuropsychiatric Inventory (caregiver version) test results (NPI / ES) (total score);
- Result of Mini-GDS (total score);
- Results of the QOL-AD (total score);
- IADL results

Regarding drug treatments, the number and percentage of subjects taking neuroleptics, benzodiazepines, anxiolytics, antidepressants, hypnotics or thymo-regulators at inclusion will be described.

6.5.4 Description of the data collected during the first multidisciplinary team meeting (MDTM1)

This descriptive analysis concerns the subjects in the intervention group only.

Will be described:

- The number and percentage of recommendations proposed during MDTM1 that were followed by type of pathology;
- The number and percentage of pathologies that have been treated;
- The number and percentage of pathologies that have been resolved;
- The reasons why the recommendations were not followed;
- For diagnoses of dementia, the number and percentage of families and health care teams informed;
- The presence of new elements by type;
- The presence of new recommendations.

6.5.5 Description of the data collected during the follow-up

The number and percentage of subjects per group who had at least one hospitalization during their follow-up period will be described for each hospitalization service (ED, intensive care unit, medicine, surgery, geriatrics and other), for each type of hospitalization (planned or in emergency).

The number and percentage of subjects per group having had at least one specialized consultation during their follow-up period will be described globally and then for each type of consultation (within a hospital, clinic, practice or other) and for the following specialties: psychiatry, neurology and geriatrics.

6.5.6 Description of the data collected at the end-of-study visit

In addition to the already detailed endpoints, the following characteristics of the topics identified during the end-of-study visit will be described:

- Time between the inclusion visit and the end-of-study visit in months;
- Overall MMSE score;
- Results of the clock test (score and normality);
- Results of the Borson Mini-Cog (normality);

For the intervention group, the differences between the scores of the different scales at baseline and at the end of the study will be described as a quantitative variable (end of study score - inclusion score).

6.5.7 Study dropout

The number and percentage of subjects dropped out prematurely will be described in the total population and by group as well as their main reason for leaving study and their time of participation (<3 months, [3-6 [months, [6-9 [months, [9-12 [months, [12-15 [months,> = 15 months].

6.6 Sensitivity analyzes

As sensitivity analyzes, the main analysis on the primary endpoint at 1 year will be conducted on the PP1 population. The 18-month primary endpoint analysis will be done on PP1 and PP2 populations in addition to the ITT analysis.

6.7 Analysis in subgroups

To determine if the effect of the intervention is different in NH that have an Alzheimer's unit and in those that do not have, the variable "presence of an Alzheimer's unit yes / no" will be added in the model of principal analysis and its interaction with the group. If the interaction is significant at 20%, the model will be presented separately for NHs with Alzheimer's unit and those without.

The same procedure will be made for public or private NHs.

7 MISSING DATA MANAGEMENT

In general, no imputation method will be applied to missing data.

Regarding incomplete dates, an in-depth study will be done on the dates used to calculate the aforementioned deadlines. For these dates, if only the day is missing it will be imputed by the value 15. No imputation will be made if the month or the year are missing. The dates that have been imputed will be listed in the statistical analysis report.

8 STATISTICAL SOFTWARE USED

All statistical analyzes detailed in this plan will be conducted using SAS® software (SAS Institute Inc., Cary NC) version 9.4 or higher.

Signature of the statistical analysis plan

FUNCTION	NAME	SIGNATURE	DATE
**Principal Investigator**	**Pr Yves Rolland**<br>Gérontopôle de Toulouse<br>La Cité de la Santé<br>Hôpital La Grave, 1 place Lange<br>31300 Toulouse<br>Tél : 05 61 77 70 21<br>Télécopie : 05 61 77 64 75<br>Courriel : rolland.y@chu-toulouse.fr		07/22/2016
**Project manager**	**Dr Neda Tavassoli**<br>Gérontopôle de Toulouse<br>La Cité de la Santé<br>Hôpital La Grave, 1 place Lange<br>31300 Toulouse<br>Tél : 05 61 77 70 13<br>Télécopie : 05 61 77 64 75<br>Courriel : tavassoli.n@chu-toulouse.fr		07/22/2016
**Methodologist**	**Pr Sandrine Andrieu**<br>UMR 1027<br>Laboratoire d'épidémiologie<br>37, allées jules Guesde<br>31073 TOULOUSE cedex 7<br>Email : sandrine.andrieu@univ-tlse3.fr<br>Tel : 05 61 14 59 20		
**Biostatistician**	**Elodie Tournay**<br>Unité de Soutien Méthodologique à la recherche du CHU de Toulouse (USMR)<br>37, allées jules Guesde<br>31073 TOULOUSE cedex 7<br>Email : tournay.e@chu-toulouse.fr<br>Tel : 05 61 14 56 73		

References

- Cummings JL, Mega M, Gray K, Rosenberg-Thompson S, Carusi DA, Gornbein J. The Neuropsychiatric Inventory: comprehensive assessment of psychopathology in dementia. Neurology 1994;44:2308-2314.
- Lawton MP, Brody E. Assessment of older people : self-maintening and instrumental activities of daily living. Gerontologist, 1969;9 : 179-186.
- Ready RE, Ott BR. Integrating patient and informant reports on the Cornell-Brown Quality-of-Life Scale. Am J Alzheimers Dis Other Demen. 2007 Dec-2008 Jan;22(6):528-34.
- Robert PH, Medecin I, Vincent S, Staccini P, Cattelin F, Goni S. L'inventaire en neuropsychiatrie : validation de la version française d'un instrument destiné à évaluer les troubles du comportement chez les patients déments. Maladie d'Alzheimer vol 5 , 1998, 63-88.
- Rolland Y et al. Descriptive study of nursing home residents from the REHPA network. J Nutr Health Aging. 2009 Oct;13(8):679-83.
