# Supplementary material for: Systematic Dementia Screening by Multidisciplinary Team Meetings in Nursing Homes for Reducing Emergency Department Transfers: The IDEM Cluster Randomized Clinical Trial
Source: JAMA Netw Open. 2020 Feb 26;3(2):e200049. doi: 10.1001/jamanetworkopen.2020.0049 (PMC7137681; doi:10.1001/jamanetworkopen.2020.0049)
Supplement: Supplement 2. — eAppendix 1. Role of Coordinating Physician in NH eAppendix 2. Stratification Criteria of Cluster Randomization eAppendix 3. GIR Score eAppendix 4. Inclusion and Visits in NHs eAppendix 5. Content of MDTM eAppendix 6. Classification of Hospitalizations as Appropriate or Inappropriate eAppendix 7. Per-protocol Analyses eAppendix 8. Exploratory Analyses eAppendix 9. Results of Per-protocol Analyses eAppendix 10. Results of Exploratory Analyses eReferences. eFigure. Study Flow-chart eTable 1. Comprehensive Geriatric Assessment at Inclusion and End of Study Visits (mITT Population) eTable 2. Assessment of Intervention Effect on Primary and Secondary Endpoints Observed in the 64 Nursing Homes in Person-Years (mITT Population, N=1401) eTable 3. Estimation and Test of Effect of Dementia Diagnosis on Risk of ED Transfer During 12-Month Follow-up (mITT Population) (N=1401) eTable 4. Assessment of Effect of Following the Recommendations of the First MDTM by GP on Risk of ED Transfer During 12-Month and 18-Month Follow-up (Subjects With MDTM1 Recommendations Followed by GPs Versus Control) (N=1086) eTable 5. Assessment of Effect of Following the Recommendations of the First and the Second MDTMs by GP on Risk of ED Transfer During 18-Month Follow-up (Subjects With Two MDTMs Recommendations Followed by GPs Versus Control) (N=960) eTable 6. Assessment of Effect of Following the Recommendations of the First MDTM by GP on Risk of ED Transfer During 12-Month and 18-Month Follow-up (Subjects With MDTM1 Recommendations Followed by GPs Versus Those With MDTM1 Recommendations Not Followed by GPs) (N=445) eTable 7. Assessment of Effect of Following the Recommendations of the First and the Second MDTMs by GP on Risk of ED Transfer During 18-Month Follow-up (Subjects With Two MDTMs Recommendations Followed by GPs Versus Those With Two MDTMs Recommendations Not Followed by GPs) (N=371) [file jamanetwopen-3-e200049-s002.pdf]

Supplementary Online Content

Rolland Y, Tavassoli N, de Souto Barreto P, et al. Systematic dementia screening by multidisciplinary team meetings in nursing homes for reducing emergency department transfers: the IDEM cluster randomized clinical trial. *JAMA Netw Open.* 2020;3(2):e200049. 10.1001/jamanetworkopen.2020.0049

eAppendix 1. Role of Coordinating Physician in NH
eAppendix 2. Stratification Criteria of Cluster Randomization
eAppendix 3. GIR Score
eAppendix 4. Inclusion and Visits in NHs
eAppendix 5. Content of MDTM
eAppendix 6. Classification of Hospitalizations as Appropriate or Inappropriate
eAppendix 7. Per-protocol Analyses
eAppendix 8. Exploratory Analyses
eAppendix 9. Results of Per-protocol Analyses
eAppendix 10. Results of Exploratory Analyses
eReferences.
eFigure. Study Flow-chart
eTable 1. Comprehensive Geriatric Assessment at Inclusion and End of Study Visits (mITT Population)
eTable 2. Assessment of Intervention Effect on Primary and Secondary Endpoints Observed in the 64 Nursing Homes in Person-Years (mITT Population, N=1401)
eTable 3. Estimation and Test of Effect of Dementia Diagnosis on Risk of ED Transfer During 12-Month Follow-up (mITT Population) (N=1401)
eTable 4. Assessment of Effect of Following the Recommendations of the First MDTM by GP on Risk of ED Transfer During 12-Month and 18-Month Follow-up (Subjects With MDTM1 Recommendations Followed by GPs Versus Control) (N=1086)
eTable 5. Assessment of Effect of Following the Recommendations of the First and the Second MDTMs by GP on Risk of ED Transfer During 18-Month Follow-up (Subjects With Two MDTMs Recommendations Followed by GPs Versus Control) (N=960)
eTable 6. Assessment of Effect of Following the Recommendations of the First MDTM by GP on Risk of ED Transfer During 12-Month and 18-Month Follow-up (Subjects With MDTM1 Recommendations Followed by GPs Versus Those With MDTM1 Recommendations Not Followed by GPs) (N=445)
eTable 7. Assessment of Effect of Following the Recommendations of the First and the Second MDTMs by GP on Risk of ED Transfer During 18-Month Follow-up (Subjects With Two MDTMs Recommendations Followed by GPs Versus Those With Two MDTMs Recommendations Not Followed by GPs) (N=371)

This supplementary material has been provided by the authors to give readers additional information about their work.

© 2020 Rolland Y et al. *JAMA Netw Open.*

eAppendix 1: Role of coordinating physician in NH

In France, each registered NH is required to have a coordinating physician among its staff members. The coordinating physician is a geriatrician who has received at least 140 hours of geriatric training (70 hours of theoretical and 70 hours of practical courses). He/she is responsible for the comprehensive geriatric assessment (CGA) of each resident and for coordination of healthcare in the NH. Treatments including drug prescription or the decision to hospitalise the patient remain under the responsibility of the resident's general practitioner (GP).

eAppendix 2: Stratification criteria of cluster randomisation

The permuted blocks of two were stratified by the presence or absence of a specialised Alzheimer's care unit in the NH, participation or non-participation of the NH's coordinating physician in the REHPA geriatric network congresses (an NH research network in the Toulouse area, France) where important information on the IDEM study was diffused, and the NH dependence score (weighted mean dependence level, or groupe iso-ressource moyen pondéré, GMP)[1] below or above the median (range from 70 to 1000, median 716). The GMP, a funding indicator for French NHs, scores the average level of dependence of NH residents. The higher the GMP score, the greater the public funding allocated to the NH.

eAppendix 3: GIR score

French level of dependence score from 1 to 6 (1 = completely dependent or bedridden, 6 = completely independent).

eAppendix 4: Inclusion and visits in NHs

After a 3-month preselection period to identify NH residents meeting the study criteria, the preselected residents in both groups had an inclusion visit in the NH carried out by the coordinating physician. Each NH included all of its eligible residents over a period of two months. Sociodemographic and medical data [medical history, cognitive history, family history of dementia, hospital admissions in the past three months, pharmacological and non-pharmacological treatments, advanced care planning and level of dependence (GIR questionnaire)] were collected in both groups. The residents in the intervention group also underwent a comprehensive geriatric assessment (CGA) using validated scales: cognitive assessment

© 2020 Rolland Y et al. *JAMA Netw Open.*

[Mini-Mental State Examination (MMSE)[2], clock-drawing test,[3] Borson's Mini-Cog,[4] 5-word test,[5] test of categorical verbal fluency[6]]; functional and physical assessment [Instrumental Activities of Daily Living, (IADL)[7], one-leg standing balance test[8]]; nutritional assessment [Mini-Nutritional Assessment (MNA)[9]]; psychological assessment [Confusion Assessment Method (CAM),[10] Neuropsychiatric Inventory caregiver version (NPI),[11] Mini Geriatric Depression Scale (mini-GDS),[12] Quality of Life–Alzheimer's Disease (QOL-AD)[13]].

At the end of the 18-month follow-up, all residents in both groups underwent a final visit in the NH with the coordinating physician. Advanced care planning was noted and CGA (MMSE, clock-drawing test, Borson's Mini-Cog, IADL, GIR, NPI, QOL-AD) was carried out.

eAppendix 5: Content of MDTM

During the MDTM, the resident's medical history, results of the comprehensive geriatric assessment performed by the coordinating physician, medication and non-pharmacological treatment were systematically reviewed and discussed. The coordinating physicians organised the meetings, to which all residents' GPs were invited.

The MDTMs were organised in line with international recommendations and with the guidelines laid down by the French National Authority for Health (HAS).[14] At least one expert physician in the field of dementia (memory expert from a memory clinic) as well as the coordinating physician was required to be present. The aim of the first MDTM was to provide a multidisciplinary diagnosis based on the DSM-IV.[15] A personalised resident-centred care plan based on international guidelines and the French HAS[16] practice guidelines was defined during the MDTM. The aim of the second MDTM was to follow the resident's care plan proposed at the first meeting, to make any necessary adjustments or changes and to find ways of overcoming any difficulties that prevented its implementation.

After the meeting, the coordinating physician informed the GP of the conclusions reached by sending them a copy of the written report. This report was also added to the resident's clinical chart in the NH.

eAppendix 6: Classification of hospitalizations as appropriate or inappropriate

To assess the impact of systematic dementia screening on the appropriateness of hospitalisations, hospitalisations were classified as appropriate or inappropriate using a standardised procedure. For

hospitalisations via the ED, inappropriate transfer was defined as the absence of somatic and/or psychiatric emergency conditions and/or palliative care known before decision to transfer and/or the presence of advance directives of non-hospitalisation in the resident's medical NH chart. In summary, an ED transfer was judged inappropriate in a clinical situation that could have been appropriately managed by other means than transfer. All programmed hospitalisations were considered as appropriate. Classification was blindly and independently performed by two geriatricians (YR and AP) who had access to the residents' anonymised hospital reports. Every two weeks, each of the two geriatricians separately studied the residents' reports and gave his/her opinion on the appropriateness or inappropriateness of hospitalisation based on the standardised checklist procedure. Individual and independent rating divergences were discussed once a month in order to reach a consensus. All divergences were solved and consensus obtained.

eAppendix 7: Per-protocol analyses

Two per-protocol populations (PP1 and PP2) were used for supportive analyses of efficacy endpoints. The per-protocol populations contained all residents in the mITT population who fulfilled all eligibility criteria: the PP1 population contained those who were included in the first MDTM, and the PP2 population those who were included in both MDTMs.

eAppendix 8: Exploratory analyses

For exploratory analysis, the variables of presence of an Alzheimer's unit in the NH and its interaction with the group were added in the random logistic regression model used for the primary efficacy analysis. If the interaction term was significant at a 20% level, the effect of intervention on the primary endpoint was estimated in both subgroup populations. The same analysis was done for NH ownership (public or private).

eAppendix 9: Results of per-protocol analyses

Analysis of the primary endpoint in the PP1 population did not show any significant difference between the two groups (OR 1.34, 95% CI 0.84-2.13; p=0.22) (table 4). Per protocol analyses of the secondary endpoint of ED transfer during the entire 18-month follow-up showed no significant differences between groups in both PP1 and PP2 populations (table 4).

© 2020 Rolland Y et al. *JAMA Netw Open.*

eAppendix 10: Results of exploratory analyses

Subgroup analyses for the endpoint showed that intervention significantly increased the risk of ED transfer in NHs without an Alzheimer's special care unit (table 4). Analysis according to NH ownership provided non-significant results.

References

1. Circulaire interministérielle DGCS/SD3/DSS/SD1 n° 2013/418 du 6 décembre 2013 relative à la mise en œuvre du décret n° 2013-22 du 8 janvier 2013 relatif à l'évaluation et à la validation du niveau de perte d'autonomie et des besoins en soins des personnes hébergées dans les établissements d'hébergement pour personnes âgées dépendantes et fixant la composition et le fonctionnement de la commission régionale de coordination médicale mentionnée à l'article L.314-9 du code de l'action sociale et des familles. http://solidarites-sante.gouv.fr/fichiers/bo/2014/14-01/ste_20140001_0000_0074.pdf (accessed 2 March 2019).
2. Folstein MF, Folstein SE, McHugh PR. "Mini-mental state". A practical method for grading the cognitive state of patients for the clinician. *J Psychiatr Res* 1975 12:189–98.
3. Nair AK, Gavett BE, Damman M, *et al.* Clock drawing test ratings by dementia specialists: interrater reliability and diagnostic accuracy. *J Neuropsychiatry Clin Neurosci* 2010;22:85–92.
4. Borson S, Scanlan J, Brush M, Vitaliano P, Dokmak A. The mini-cog: a cognitive 'vital signs' measure for dementia screening in multi-lingual elderly. *Int J Geriatr Psychiatry* 2000;15:1021–7.
5. Dubois B, Touchon J, Portet F, Ousset PJ, Vellas B, Michel B. "The 5 words": a simple and sensitive test for the diagnosis of Alzheimer's disease. *Presse Med* 2002:31:1696–9.
6. Cardebat D, Doyon B, Puel M, Goulet P, Joanette Y. Formal and semantic lexical evocation in normal subjects. Performances and dynamics of production as a function of sex, age and educational level. *Acta Neurol Belg* 1990;90:207–17.
7. Lawton MP, Brody EM. Assessment of older people: self–maintaining and instrumental activities of daily living. *Gerontologist* 1969;9:179–86.
8. Vellas BJ, Rubenstein LZ, Ousset PJ, *et al.* One-leg standing balance and functional status in a population of 512 community-living elderly persons. *Aging* 1997;9:95–8.
9. Vellas B, Villars H, Abellan G, *et al.* Overview of the MNA--Its history and challenges. *J Nutr Health Aging* 2006;10:456–63.

© 2020 Rolland Y et al. *JAMA Netw Open.*

10. Inouye SK, van Dyck CH, Alessi CA, Balkin S, Siegal AP, Horwitz R. Clarifying confusion: the confusion assessment method. A new method for detection of delirium. *Ann Intern Med* 1990;113:941–8.

11. Cummings JL, Mega M, Gray K, *et al.* The Neuropsychiatric Inventory: comprehensive assessment of psychopathology in dementia. *Neurology* 1994;44:2308–14.

12. Clément JP, Nassif RF, Léger JM, Marchan F. Development and contribution to the validation of a brief French version of the Yesavage Geriatric Depression Scale. *Encephale* 1997;23:91–9.

13. Ready RE, Ott BR, Grace J. Insight and cognitive impairment: effects on quality-of-life reports from mild cognitive impairment and Alzheimer's disease patients. *Am J Alzheimers Dis Other Demen* 2006;21:242–8.

14. Une démarche de l'évaluation de la qualité : Réunion de concertation pluridisciplinaire en cancérologie. Recommandations de l'HAS. http://www.has-sante.fr/portail/upload/docs/application/pdf/2009-08/traceur_fiche_epp_rcp.pdf (accessed 2 March 2019).

15. American Psychiatric Association. *Diagnostic and statistical manual of mental disorders* (4th ed., text rev.). Washington, DC: American Psychiatric Association, 2000.

16. Maladie d'Alzheimer et maladies apparentées : diagnostic et prise en charge. Recommandation de l'HAS. Available at: http://www.has-sante.fr/portail/upload/docs/application/pdf/2011-12/recommandation_maladie_d_alzheimer_et_maladies_apparentees_diagnostic_et_prise_en_charge.pdf (accessed 2 March 2019).

© 2020 Rolland Y et al. *JAMA Netw Open.*

eFigure 1. Study flow-chart

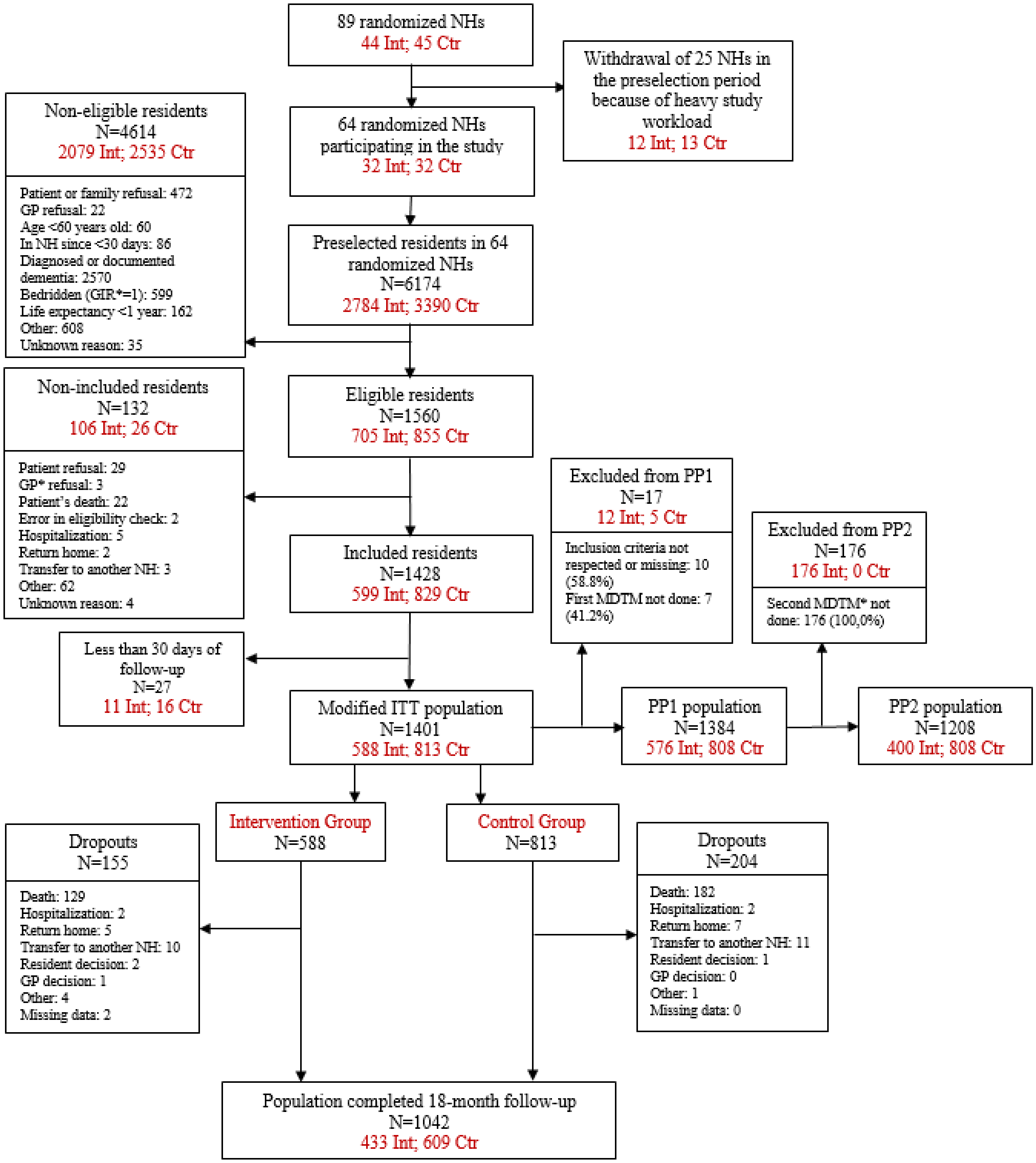

© 2020 Rolland Y et al. *JAMA Netw Open*.

Ctr, control; GP, general practitioner; GIR, Groupe iso-ressource, French level of dependence score; Int, intervention; MDTM, multidisciplinary team meetings; NH, Nursing home.

eTable 1. Comprehensive geriatric assessment at inclusion and end of study visits (mITT population)

	Inclusion visit			End of study visit			Difference between inclusion and end of study visits	
	**Intervention***	**Control***		**Intervention***	**Control***		**Intervention***	**Control***
	**(N=588)**	**(N=813)**		**(N=433)**	**(N=609)**			
Instrumental Activities of Daily Living (IADL)	2.4 ± 1.10	-		2.1 ± 1.19	2.0 ± 1.21		-0.3 ± 1.08	-
	n=580			n=435	n=599		n=432	
Mini-Mental State Examination (MMSE) (0-30)	21.8 ± 5.31	-		20.7 ± 6.51	19.8 ± 6.74		-1.5 ± 3.74	-
	n=577			n=405	n=548		n=404	
Clock test (0-7)	2.8 ± 2.45	-		3.0 ± 2.57	3.3 ± 2.67		-0.1 ± 2.27	-
	n=295			n=247	n=304		n=150	
Clock test normal score (=7)	32 (10.8)	-		36 (14.6)	55 (18.0)		-	-
	n=295			n=247	n=305			
Borson's Mini-Cog normal score	69 (23.2)	-		83 (33.7)	78 (25.5)		-	-
	n=297			n=246	n=306			
Five-word test: global score	8.0 ± 2.30	-	-	-	-	-	-	-
	n=319							
Five-word test: learning score	4.4 ± 0.99	-	-	-	-	-	-	-
	n=320							
Five-word test: memory score	3.7 ± 1.55	-	-	-	-	-	-	-
	n=319							
Verbal fluency test (CNT)		-	-	-	-	-	-	-

© 2020 Rolland Y et al. *JAMA Netw Open.*

Total number of words generated	13.4 ± 6.31 n=328	-	-	-	-	-	-	-
Total number of acceptable words	12.1 ± 5.82 n=327	-	-	-	-	-	-	-
Confusion Assessment Method (CAM): acute confusional status at inclusion	1 ± 0.2 n=576	-	-	-	-	-	-	-
Mini Nutritional Assessment (MNA): global score	22.9 ± 3.64 n=576	-	-	-	-	-	-	-
Risk of malnutrition (score between 17 and 23.5)	250 (43.4) n=576	-	-	-	-	-	-	-
Poor nutritional status (score <17)	36 (6.3) n=576	-	-	-	-	-	-	-
Abnormal one-leg standing test	443 (79.8) n=555	-	-	-	-	-	-	-
High probability of depression (Mini-GDS score ≥1)	385 (66.8) n=576	-	-	-	-	-	-	-

*n (%) or mean ± SD

CNT, Contingency Naming Test, GDS, Geriatric Depression Scale, mITT, modified intention to treat, SD, standard deviation.

© 2020 Rolland Y et al. *JAMA Netw Open.*

eTable 2. Assessment of intervention effect on primary and secondary endpoints observed in the 64 nursing homes in person-years (mITT population, N=1401)

	**Intervention* (N=32)**	**Control* (N=32)**	**All (N=64)**
ED admission during 12-month follow-up	15.7 ± 13.04 [0.0; 58.8]	12.9 ± 9.53 [0.0; 39.3]	14.3 ± 11.42 [0.0; 58.8]
Incidence rates of ED admission during 12-month follow-up for 100 person-years	22.1 ± 21.04 [0.0; 108.2]	18.7 ± 16.64 [0.0; 64.6]	20.4 ± 18.90 [0.0; 108.2]
Incidence rates of ED admission during 18-month follow-up for 100 person-years	21.3 ± 19.25 [0.0; 85.8]	20.0 ± 17.53 [0.0; 74.3]	20.7 ± 18.27 [0.0; 85.8]
Mean number of work interruptions in the staff during 18-month follow-up for 100 residents	168.2 ± 172.11 [25.3; 916.1]	124.7 ± 142.30 [7.4; 810.0]	146.1 ± 157.92 [7.4; 916.1]
Mean number of days of work interruptions in the staff during 18-month follow-up for 100 residents	1599.7 ± 1097.76 [352.2; 4904.5]	1150.4 ± 944.14 [8.0; 4058.4]	1371.5 ± 1039.27 [8.0; 4904.5]
Mean number of work interruptions in the staff per month during 18-month follow-up for 100 residents	9.3 ± 9.56 [1.4; 50.9]	6.9 ± 7.91 [0.4; 45.0]	8.1 ± 8.77 [0.4; 50.9]
Mean number of days of work interruptions in the staff per month during 18-month follow-up for 100 residents	88.9 ± 60.99 [19.6; 272.5]	63.9 ± 52.45 [0.4; 225.5]	76.2 ± 57.74 [0.4; 272.5]

*Mean ± SD [min; max]

ED, emergency department, mITT, modified intention to treat.

© 2020 Rolland Y et al. *JAMA Netw Open.*

eTable 3. Estimation and test of effect of dementia diagnosis on risk of ED transfer during 12-month follow-up (mITT population) (N=1401).

	**No diagnosis of dementia (N=1165)**	**Diagnosis of dementia at first MDTM (N=236)**	**OR**	**95% CI**	**p Value**
Primary endpoint: ED transfer during 12-month follow-up§	159 (13.6%)	40 (16.9%)	1.14	[0.73 to 1.77]	0.56

§The ICC, indicating the similarity between measurements of participants from the same cluster and those from different clusters, was estimated at 0.1129 for the primary endpoint.

CI, confidence interval, ED, emergency department, MDTM, MultiDisciplinary Team Meetings, mITT, modified intention to treat, OR, odds ratio.

© 2020 Rolland Y et al. *JAMA Netw Open.*

eTable 4. Assessment of effect of following the recommendations of the first MDTM by GP on risk of ED transfer during 12-month and 18-month follow-up (subjects with MDTM1 recommendations followed by GPs versus control) (N=1086).

	Resident's GP followed the recommendations of the first MDTM (N=273)	Control (N=813)	OR* / RR**	95% CI	p Value
Primary endpoint: ED transfer during 12-month follow-up§	40 (14.7%)	104 (12.8%)	1.29*	[0.73 to 2.27]	0.38
Incidence rate of ED transfer during 12-month follow-up for 100 person-years	19.21 [11.97-30.84]	15.93 [11.11-22.85]	1.21**	[0.68 to 2.15]	0.52
ED transfer during 18-month follow-up	47 (17.2%)	145 (17.8%)	1.04*	[0.58 to 1.88]	0.88
Incidence rate of ED transfer during 18-month follow-up for 100 person-years	16.81 [10.57-26.74]	16.59 [11.68-23.57]	1.01**	[0.57 to 1.79]	0.96

§The ICC, indicating the similarity between measurements of participants from the same cluster and those from different clusters, was estimated at 0.1129 for the primary endpoint.

*OR (Intervention vs. Control)

**RR (Intervention/Control)

CI, confidence interval, ED, emergency department, GP, general practitioner, MDTM, MultiDisciplinary Team Meetings, OR, odds ratio, RR, rate ratio; vs., versus.

© 2020 Rolland Y et al. *JAMA Netw Open.*

eTable 5. Assessment of effect of following the recommendations of the first and the second MDTMs by GP on risk of ED transfer during 18-month follow-up (subjects with two MDTMs recommendations followed by GPs versus control) (N=960).

	**Resident's GP followed the recommendations of the first and the second MDTMs (N=147)**	**Control (N=813)**	**OR* / RR****	**95% CI**	**p Value**
ED transfer during 18-month follow-up	21 (14.3%)	145 (17.8%)	0.78*	[0.37 to 1.63]	0.50
Incidence rate of ED transfer during 18-month follow-up for 100 person-years	10.82 [5.75-20.36]	16.51 [11.61-23.50]	0.66**	[0.32 to 1.33]	0.24

*OR (Intervention vs. Control)

**RR (Intervention/Control)

CI, confidence interval, ED, emergency department, GP, general practitioner, MDTM, MultiDisciplinary Team Meetings, OR, odds ratio, RR, rate ratio; vs., versus.

© 2020 Rolland Y et al. *JAMA Netw Open.*

eTable 6. Assessment of effect of following the recommendations of the first MDTM by GP on risk of ED transfer during 12-month and 18-month follow-up (subjects with MDTM1 recommendations followed by GPs versus those with MDTM1 recommendations not followed by GPs) (N=445).

	**Resident's GP followed the recommendations of the first MDTM (N=273)**	**Resident's GP did not follow the recommendations of the first MDTM (N=172)**	**OR* / RR****	**95% CI**	**p Value**
Primary endpoint: ED transfer during 12-month follow-up§	40 (14.7%)	33 (19.2%)	0.82*	[0.46 to 1.46]	0.50
Incidence rate of ED transfer during 12-month follow-up for 100 person-years	20.27 [13.73-29.92]	20.75 [12.88-33.42]	0.98**	[0.57 to 1.67]	0.93
ED transfer during 18-month follow-up	47 (17.2%)	47 (27.3%)	0.63*	[0.37 to 1.09]	0.09
Incidence rate of ED transfer during 18-month follow-up for 100 person-years	17.90 [12.30-26.05]	21.80 [13.97-34.03]	0.82**	[0.50 to 1.34]	0.42

§The ICC, indicating the similarity between measurements of participants from the same cluster and those from different clusters, was estimated at 0.1129 for the primary endpoint.

*OR (Intervention vs. Control)

**RR (Intervention/Control)

CI, confidence interval, ED, emergency department, GP, general practitioner, MDTM, MultiDisciplinary Team Meetings, OR, odds ratio, RR, rate ratio; vs., versus.

© 2020 Rolland Y et al. *JAMA Netw Open.*

eTable 7. Assessment of effect of following the recommendations of the first and the second MDTMs by GP on risk of ED transfer during 18-month follow-up (subjects with two MDTMs recommendations followed by GPs versus those with two MDTMs recommendations not followed by GPs) (N=371).

	**Resident's GP followed the recommendations of the first MDTM (N=147)**	**Resident's GP did not follow the recommendations of the first MDTM (N=224)**	**OR* / RR***	**95% CI**	**p Value**
ED transfer during 18-month follow-up	21 (14.3%)	56 (25.0%)	0.56*	[0.30 to 1.08]	0.08
Incidence rate of ED transfer during 18-month follow-up for 100 person-years	12.69 [7.77-20.74]	20.60 [13.84-30.66]	0.62**	[0.35 to 1.08]	0.09

*OR (Intervention vs. Control)

**RR (Intervention/Control)

CI, confidence interval, ED, emergency department, GP, general practitioner, MDTM, MultiDisciplinary Team Meetings, OR, odds ratio, RR, rate ratio; vs., versus.

© 2020 Rolland Y et al. *JAMA Netw Open.*
