# Supplementary material for: Systematic Dementia Screening by Multidisciplinary Team Meetings in Nursing Homes for Reducing Emergency Department Transfers: The IDEM Cluster Randomized Clinical Trial
Source: JAMA Netw Open. 2020 Feb 26;3(2):e200049. doi: 10.1001/jamanetworkopen.2020.0049 (PMC7137681; doi:10.1001/jamanetworkopen.2020.0049)
Supplement: Supplement 3. — Data Sharing Statement [file jamanetwopen-3-e200049-s003.pdf]

Rolland Y, Tavassoli N, de Souto Barreto P, et al. Systematic dementia screening by multidisciplinary team meetings in nursing homes for reducing emergency department transfers: the IDEM cluster randomized clinical trial. *JAMA Netw Open*. 2020;3(2):e200049. 10.1001/jamanetworkopen.2020.0049

Data Sharing Statement

Data
Data available: *No*

© 2020 Rolland Y et al. *JAMA Network Open.*
